# Gene Expression Patterns Analysis in the Supraspinatus Muscle after a Rotator Cuff Tear in a Mouse Model

**DOI:** 10.1155/2018/5859013

**Published:** 2018-12-23

**Authors:** Yong-Soo Lee, Ja-Yeon Kim, Hyo-Nam Kim, Dhong-Won Lee, Seok Won Chung

**Affiliations:** Department of Orthopedic Surgery, Konkuk University School of Medicine, Seoul, Republic of Korea

## Abstract

Rotator cuff tear is a muscle-tendinous injury representative of various musculoskeletal disorders. In general, rotator cuff tear occurs in the tendon, but it causes unloading of the muscle resulting in muscle degeneration including fatty infiltration. These muscle degenerations lead to muscle weakness, pain, and loss of shoulder function and are well known as important factors for poor functional outcome after rotator cuff repair. Given that rotator cuff tear in various animal species results in similar pathological changes seen in humans, the animal model can be considered a good approach to understand the many aspects of the molecular changes in injured muscle. To comprehensively analyze changes in gene expression with time following a rotator cuff tear, we established a rotator cuff tear in mouse supraspinatus tendon of shoulder. At weeks 1 and 4 after the tear, the injured muscles were harvested for RNA isolation, and microarray analysis was performed. Expression patterns of genes belonging to 10 muscle physiology-related categories, including aging, apoptosis, atrophy, and fatty acid transport, were analyzed and further validated using real-time PCR. A total of 39,429 genes were analyzed, and significant changes in expression were observed for 12,178 genes at 1 week and 2,370 genes at 4 weeks after the tear. From the list of top 10 significantly up- and downregulated genes at the 2 time periods and the network evaluation of relevant genes according to the 10 categories, several important genes in each category were observed. In this study, we found that various genes are significantly altered after rotator cuff tear, and these genes may play key roles in controlling muscle degeneration after a rotator cuff tear.

## 1. Introduction

Rotator cuff tear is a common condition that causes pain and functional disability, and it has been reported that more than 50% of patients over the age of 60 years have a rotator cuff tear [1]. Surgical repair of rotator cuff tears is widely practiced and has been a commonly accepted treatment for full-thickness rotator cuff tears, especially when conservative treatment fails [2]. However, failure in rotator cuff healing remains one of the most common and well-known complications of surgical repair [3, 4]. Several factors associated with rotator cuff muscle changes, such as aging [5], apoptosis [6], muscle degeneration [7], sarcopenia [8], muscle atrophy [9], and muscle fatty infiltration [10], have been demonstrated to be associated with rotator cuff tear. Further, these anatomical and physiological rotator cuff muscle changes reportedly result in healing failure and poor functional outcomes after rotator cuff repair [11–13]. Various trials have been performed to improve the quality of rotator cuff muscles by using growth factors, platelet-rich plasma, stem cells or its secretome, and anabolic steroids [14–19]; however, only a few studies to identify the molecular mechanisms underlying changes in the rotator cuff muscle after a rotator cuff tear have been performed [10, 20–22].

Identification of gene expression patterns of rotator cuff muscle cells after a rotator cuff tear would be the first step and an important determinant in understanding the rotator cuff tear-related muscle changes and improving outcomes of rotator cuff repair surgery. Several studies have suggested various causes and mechanisms for muscle changes, such as atrophy and degeneration [9, 10, 21–25]; however, despite this, much remains unknown. Progression of muscle degeneration, atrophy, and fatty infiltration is usually caused by abnormal signaling processes in muscle cells [25]. Abnormal muscle cell activities are the results of external stimuli, such as physical damage or aging, which lead to differentiation into fat cells or fibrous tissue and ultimately myocyte destruction (instead of normal myocyte differentiation) [26]. Most of these cellular activities are directly related to intracellular gene regulation. Therefore, if we know how genes are regulated in muscle cells in response to external stimuli, it would be possible to understand the mechanism(s) underlying pathologic rotator cuff muscle changes after a rotator cuff tear. Further, this may also provide important information for the treatment of rotator cuff tears by controlling the expression of relevant genes. To date, only a few genes, such as those coding for peroxisome proliferator-activated receptors gamma (PPAR*γ*), CCAAT-enhancer-binding proteins alpha (CEBP*α*), myogenin, myostatin, and matrix metallopeptidases (MMPs), have been identified in relation to muscle changes after rotator cuff tears [20, 21, 24, 27]. However, to the best of our knowledge, a comprehensive analysis of the time-dependent changes in gene expression patterns after rotator cuff tears has not been reported.

Therefore, this study aimed to comprehensively analyze the patterns of gene expression in rotator cuff muscles with time after a rotator cuff tear (acute or chronic) by categorizing muscle physiology-related genes in a mouse model. In this study, we hypothesized that rotator cuff injury may cause alterations in gene expression regarding pathophysiology of rotator cuff muscle. Our study may improve understanding of molecular events after rotator cuff injury and may help the identification of novel regulation or control way to overcome the poor functional outcome after rotator cuff repair.

## 2. Materials and Methods

### 2.1. Animal Experiment

Eight-week-old male C57BL/6 mice (Orient Bio Inc., Seongnam, Korea) were used in this study. Before beginning the experiments, the mice were acclimatized to a 12:12-h light/dark cycle at 22 ± 2°C for 1 week and allowed unlimited access to food and water.

We generated the rotator cuff tear model in mice as described previously [10]. Briefly, the supraspinatus tendon of the right shoulder of each mouse was fully exposed and completely transected from the greater tuberosity of the humerus under anesthesia using Zoletile (30 mg/kg; Virbac, Carros, France) and Rompun (10 mg/kg; Bayer Korea Ltd., Seoul, Korea). The supraspinatus of the left shoulder served as a control. A 3-0 nylon suture was used to close the skin, and the mice were allowed unrestricted cage activity. At weeks 1 and 4 after the surgery, mice (n = 4 per time interval) were sacrificed by cervical dislocation, and the supraspinatus muscles of both shoulders were completely harvested from the scapular fossa. The muscle tissue samples were used for total RNA extraction. All animal experiments were approved by the Institutional Animal Care and Use Committee of the Konkuk University (IACUC: KU17122) and were performed in accordance with the Guide for the Care and Use of Laboratory Animals published by the US National Institutes of Health.

### 2.2. RNA and Gene Expression Profiling

RNA quality was assessed using Agilent 2100 Bioanalyzer (Agilent Technologies, USA), and its quantity was determined using the ND-1000 spectrophotometer (NanoDrop Technologies, USA). The total RNAs from each supraspinatus muscle at different phase (1 or 4 weeks) after injury were pooled and used for microarray. Gene expression analyses were performed using the global Affymetrix GeneChip® Human Gene 2.0 ST oligonucleotide arrays. About 300 ng of each RNA sample was used for the Affymetrix procedure, as recommended by the manufacturer (http://www.affymetrix.com). Briefly, 300 ng total RNA from each sample was converted to double-strand cDNA. Using a random hexamer incorporating a T7 promoter, amplified RNA (cRNA) was generated from the double-stranded cDNA template though an IVT (*in vitro* transcription) reaction and purified with the Affymetrix Sample Cleanup Module. cDNA was synthesized by random-primed reverse transcription using a dNTP mix containing dUTP. Next, cDNA was digested using the UDG and APE 1 restriction endonucleases, and end-labeled using the terminal transferase reaction incorporating a biotinylated dideoxynucleotide. The fragmented end-labeled cDNA was hybridized to the GeneChip® Human Gene 2.0 ST arrays for 16 h at 45°C and 60 rpm, as described in the Gene Chip Whole Transcript (WT) Sense Target Labeling Assay manual (Affymetrix). After hybridization, the chips were stained using Streptavidin Phycoerythrin (SAPE), washed in Genechip Fluidics Station 450 (Affymetrix), and scanned using Genechip Array Scanner 3000 7G (Affymetrix).

Ten gene categories that have direct or indirect effects on muscle physiology in relation to rotator cuff tears were selected. The categories were as follows: (1) aging, (2) inflammation, (3) apoptosis, (4) neovascularization, (5) extracellular matrix composition, (6) myocyte differentiation, (7) myocyte proliferation, (8) cellular migration, (9) fatty acid transport, and (10) muscle atrophy. Every gene expression values were measured, and among them, those with the fold change of rotator cuff tear group/control group were more than 2 or less than 1/2 with the raw values (log 2) of more than 4 were defined as significant genes and further analyzed. Especially, the aging-, apoptosis-, fatty acid transport-, and muscle atrophy-related categories were analyzed in detail because these categories are known to be closely associated with muscle changes, such as degeneration, fatty infiltration, and atrophy, after rotator cuff tear [10, 22, 23].

### 2.3. STRING Network

**Figure 1:**
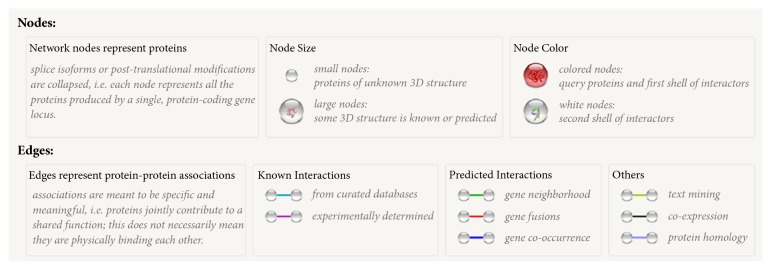
Information for interpreting the STRING network.

The genes showing significant changes in expression were selected and used as the input for STRING (Search Tool for the Retrieval of Interacting Genes/Proteins; https://string-db.org/). Protein network analyses were performed. The database and web-tool STRING is a meta-resource that integrates most of the available information on protein–protein associations, and scores, weighs, and augments it with predicted interactions, as well as with the results of automatic literature mining searches [28]. Using this, we obtained the protein interaction network images associated with functional enrichment [29]. Information regarding the size of each node and edges between nodes is shown in Figure 1 (EBIOGEN Inc., Korea).

### 2.4. Data Analysis

After the final washing and staining step, the Affymetrix GeneChip® Human Gene 2.0 ST oligonucleotide array was scanned using Affymetrix Model 3000 G7 Scanner and the image data was extracted using the Affymetrix Commnad Console software v1.1. The raw.cel file generated after the above procedure showed the expression intensity data and was used for the next step. Expression data were generated by the Affymetrix Expression Console software version 1.1. For normalization, the Robust Multi-Average (RMA) algorithm implemented in the Affymetrix Expression Console software was used. In order to find the coexpressing gene groups (which had similar expression patterns), we performed hierarchical clustering in the MultiExperiment Viewer software v4.4 (MEV; www.tm4.org). The web-based tool DAVID (Database for Annotation, Visualization, and Integrated Discovery; http://david.abcc.ncifcrf.gov/home.jsp) was used to perform biological interpretation for the differentially expressed genes. Next, these genes were classified based on the information about their functions in Gene Ontology in the KEGG Pathway database.

### 2.5. Quantitative Reverse Transcription (qRT) PCR Analysis

To validate the gene expression analysis results obtained using the microarray process described above, we performed qRT-PCR analysis for several representative genes. Total RNA was extracted from the supraspinatus muscles using the TRIzol reagent (Invitrogen, Carlsbad, CA, USA), according to the manufacturer's instructions, and used for cDNA synthesis using the Maxime RT PreMix kit (iNtRON Biotechnology, Korea). The qRT-PCR analysis was carried out using Light Cycler 480 System (Roche Diagnostics, Swiss) with 2 × qPCR BIO SyGreen Mix Lo-ROX (PCR Biosystems, London, UK). All expression data were normalized to actin expression.

### 2.6. Statistical Analysis

Descriptive statistics were used to present the analyzed data in this study.

## 3. Results

**Figure 2:**
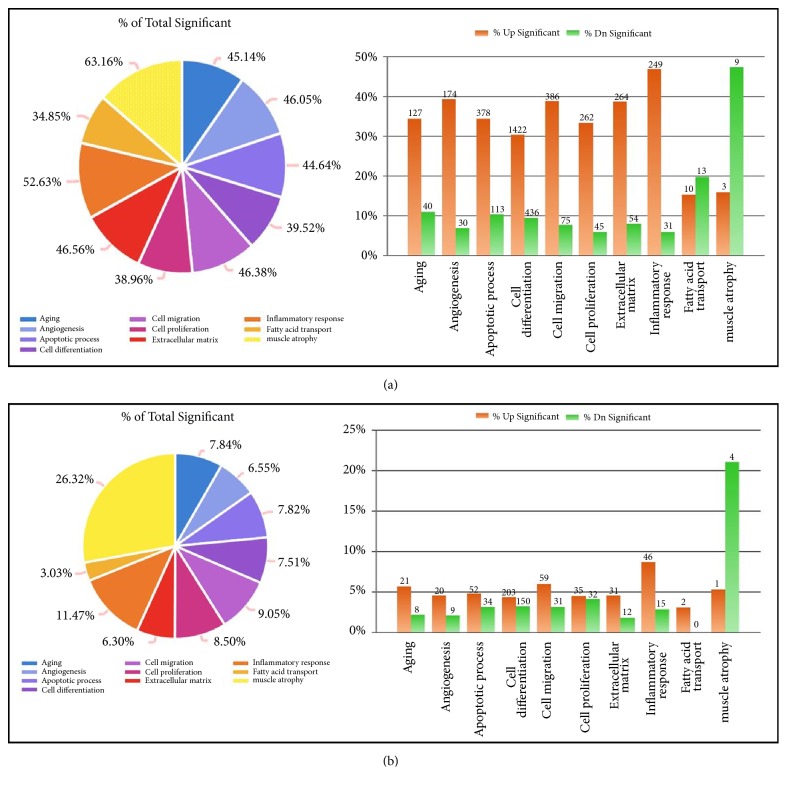
Percentages of total significant genes and up and down significant genes according to 10 categories after rotator cuff tear at (a) 1 and (b) 4 weeks.

A total of 39,429 genes were analyzed. Among these, 9,696 genes were associated with muscle physiology. Significant changes in expression were observed for 12,178 genes during the acute phase and 2,370 genes during the chronic phase. The number and distribution of genes expressed per category are shown in Figure 2 and Table 1.

**Figure 3:**
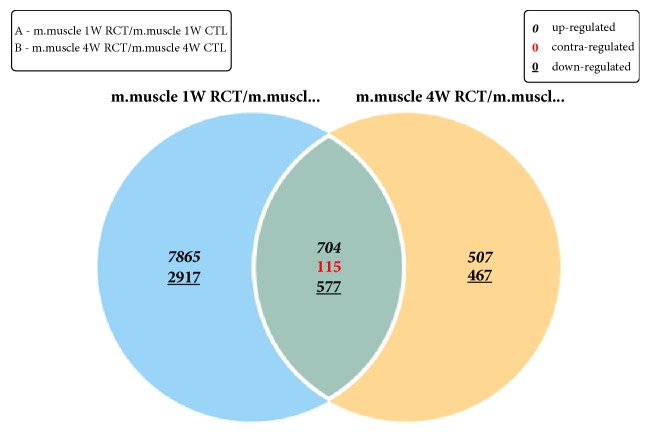
Venn diagram depicting the number of genes up-, down-, and contraregulated among the significant genes for weeks 1 and 4.

From the Venn diagram of genes showing significantly different expression at weeks 1 and 4 after the rotator cuff tear, we could identify 115 genes which showed a reverse expression pattern (genes that increased during the acute phase but decreased during the chronic phase or genes that decreased during the acute phase but increased during the chronic phase) (Figure 3).

Overall, the most highly expressed gene at week 1 was keratin 18, which increased 217.8 times compared to the control. This gene decreased 22.1 times compared to the control at week 4. Signal-regulatory protein beta 1B (Sirpb1b) and keratin 8 also displayed higher expression (more than 100 times) than the control at week 1, then subsequently decreased (expression: 4 and 16 times higher than the control, respectively) at week 4. The overall up- and downregulated genes and fold-change values of rotator cuff tear/control at weeks 1 and 4 are listed in Table 2.

### 3.1. Gene Expression Patterns in the Aging Category

**Figure 4:**
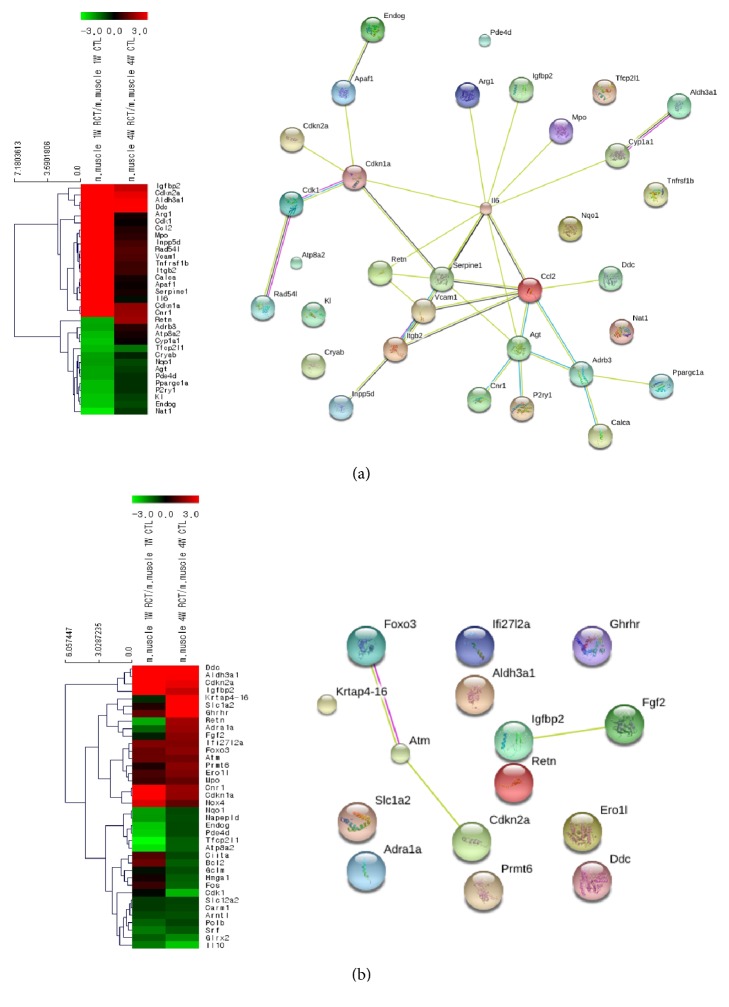
Differential expression patterns in hierarchical clustering and the STRING network for the aging category ((a) week 1 after tear; (b) week 4 after tear). Left side shows differential expression patterns and hierarchical clustering, and right side shows the STRING network for the aging category.

Analysis of the up- and downregulated genes in the 10 categories showed that insulin-like growth factor binding protein 2 (Igfbp2) was the most highly expressed gene in the aging category. It displayed 55.7 times higher expression in the RC tear side than in the control at week 1, and subsequently showed a decreasing trend (expressed 5.2 times higher than control) at week 4. In the string network which depicts the interactions between genes, we observed that IL6, Ccl2, and Vcam1, which are known to be related to inflammation or cell adhesion, actively interacted with each other showing high expression at week 1 after the RC tear. In addition, RAD54L (a DNA repair-related gene) and cyclin-dependent kinase 1 (Cdk1; a cell cycle regulation-related gene) also interacted closely (Figure 4). The top 10 up- and downregulated genes in the aging category at weeks 1 and 4 after the RC tear are listed in Table 3.

### 3.2. Gene Expression Patterns in the Apoptosis Category

**Figure 5:**
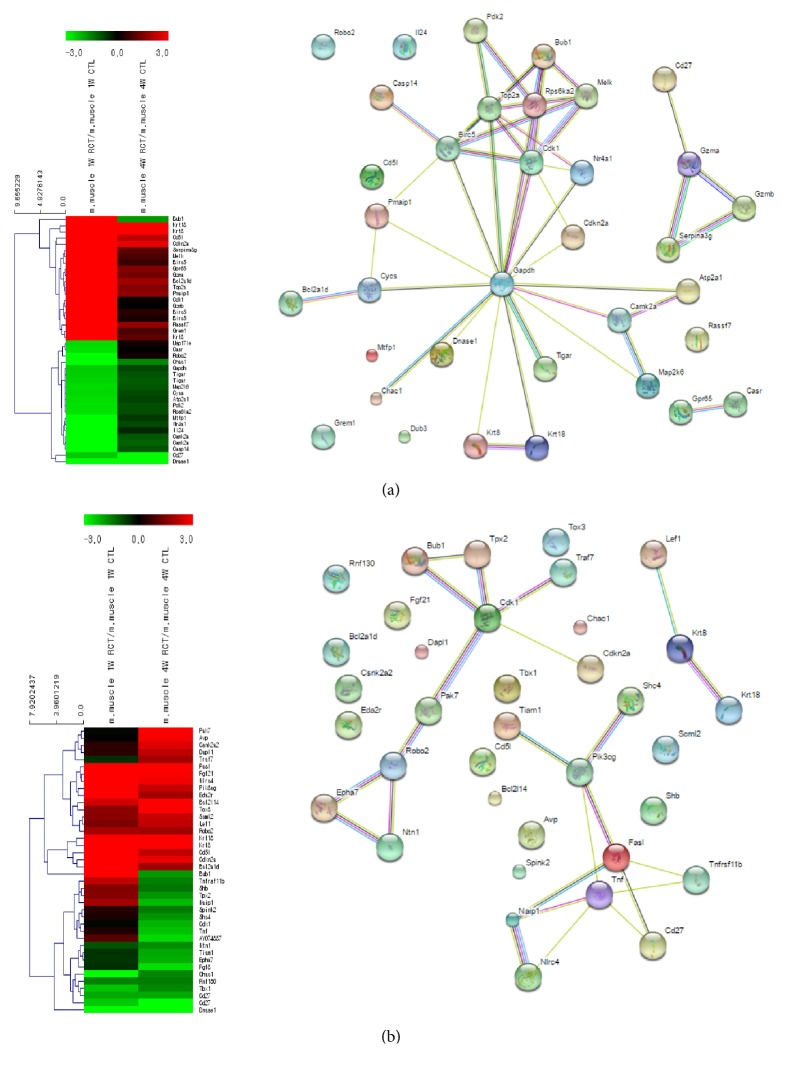
Differential expression patterns in hierarchical clustering and the STRING network for the apoptosis category ((a) week 1 after tear; (b) week 4 after tear).

In the apoptosis category, keratin 18 showed the highest expression (217.8 times higher expression than the control group) at week 1. This expression subsequently decreased (22 times higher expression than the control group) at week 4 (Figure 5 and Table 4). Interestingly, Birc5 (55.6 and 1.6 times higher expression than control at weeks 1 and 4, respectively), Rps6ka2 (0.2 times and 0.5 times higher expression than control at weeks 1 and 4, respectively), and Bub1 (37.5 and 0.3 times higher expression than control at weeks 1 and 4, respectively), which also showed reverse expression patterns, presented active interactions with each other revealed by the STRING network analysis results of week 1. These genes are known to be involved in apoptotic processes, such as cell growth, cell cycle, and cell differentiation.

### 3.3. Gene Expression Patterns in the Muscle Atrophy Category

**Figure 6:**
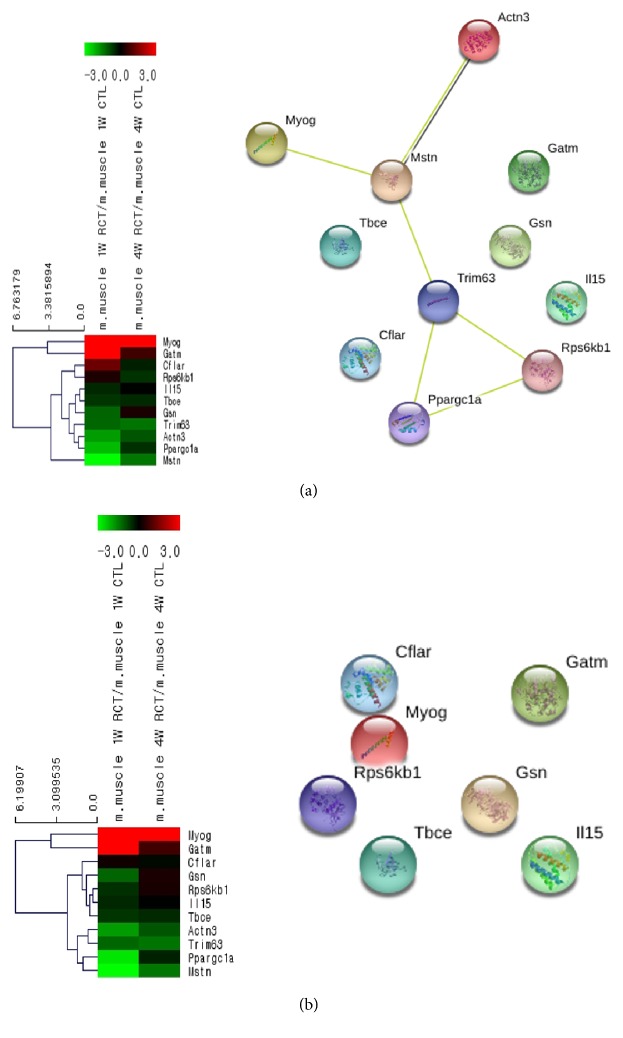
Differential expression patterns in hierarchical clustering and the STRING network for the muscle atrophy category ((a) week 1 after tear; (b) weeks 4 after tear).

Among the 39,429 genes analyzed, a total of 11 genes belonged to the muscle atrophy category (Figure 6 and Table 5). Myog (myogenin), which is associated with myogenesis, showed the highest expression at week 1. In contrast, Mstn (myostatin), which is to inhibit myogenesis, showed the lowest expression at week 1. Further, Actin 3 (actin alpha 3), which is coexpressed with myostatin, also showed low expression at week 1 but a high expression at week 4 together with myostatin. This result was in contrast to that of myogenin, which showed the highest expression at week 1 but low expression at week 4.

### 3.4. Gene Expression Patterns in the Fatty Acid Transport Category

**Figure 7:**
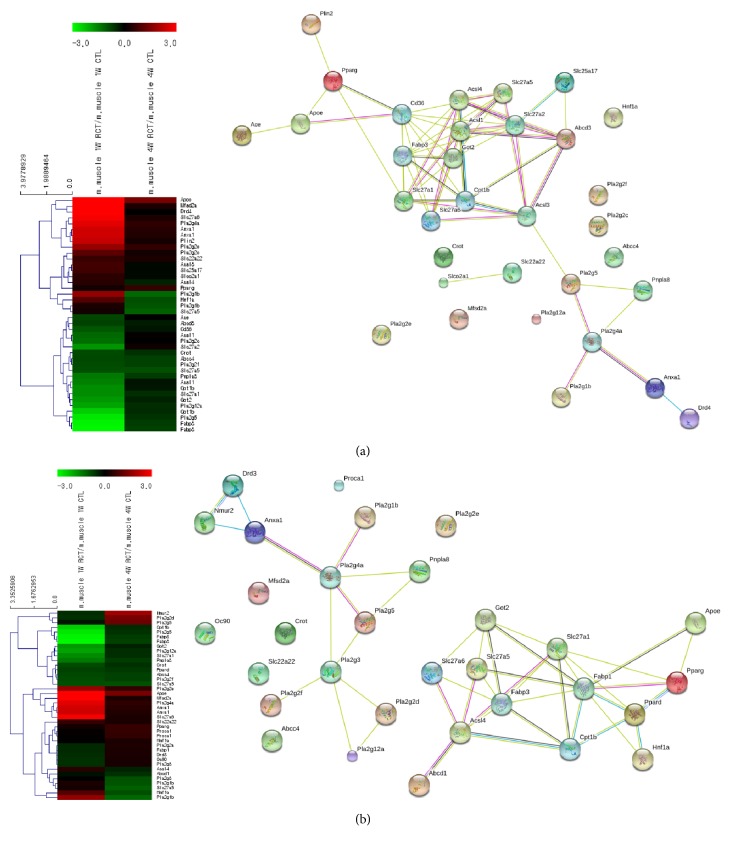
Differential expression patterns in hierarchical clustering and the STRING network for the fatty acid transport category ((a) week 1 after tear; (b) week 4 after tear).

In the fatty acid transport category, the expression of apolipoprotein E (ApoE; expressed 12.229 times higher than control at week 1), annexin A1 (expressed 5.517 times higher than control at week 1) which is a phospholipid-binding protein, and perilipin 2 (expressed 5.018 times higher than control at week 1) which is an adipose differentiation-related gene was notable. The expression of all these genes decreased at week 4 (ApoE, annexin A1, and perilipin 2 were expressed 2.729, 1.377, and 1.196 times higher than control, respectively). In addition, these genes interacted with the phospholipase groups regulating PPAR*γ* expression, which regulates fatty acid storage and glucose metabolism, at weeks 1 and 4 as shown in the STRING network (Figure 7 and Table 6).

### 3.5. Gene Expression Patterns in Other Categories Related to Muscle Physiology

Gene expression patterns and STRING networks for other categories, such as angiogenesis, inflammation, cell migration, cell proliferation, extracellular matrix, and cell differentiation, are described in ‘Supplementary Materials (available here)'.

### 3.6. Validation of Gene Expression Using Real-Time PCR Analysis

**Figure 8:**
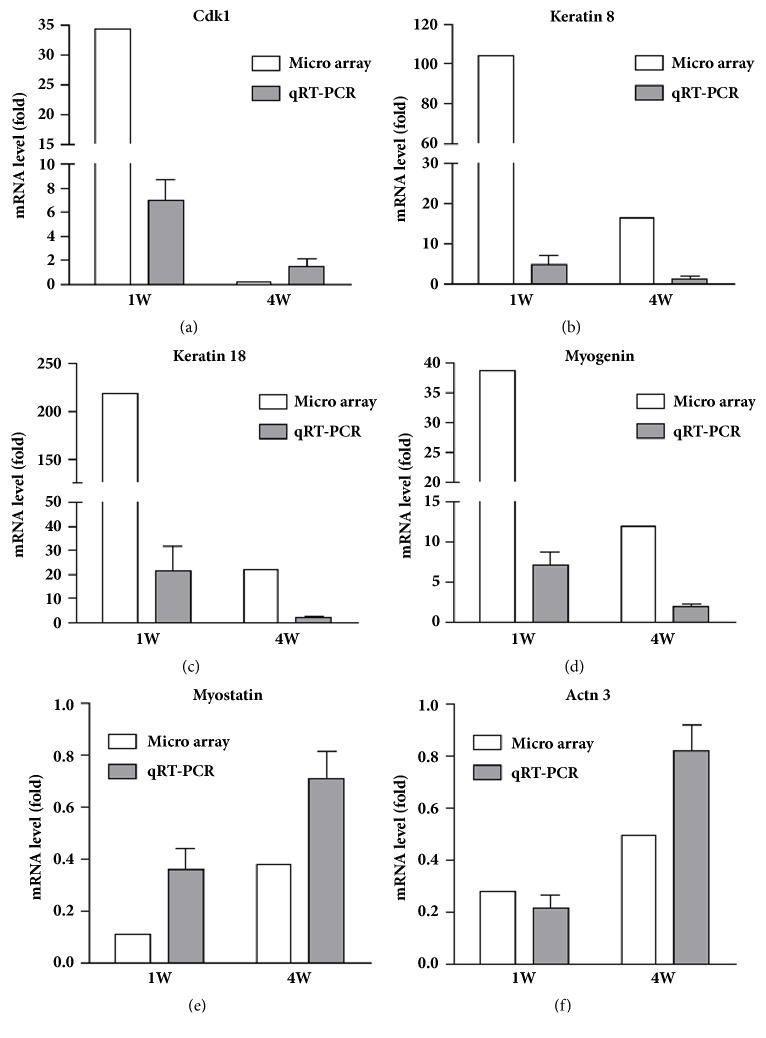
Validation of microarray gene expression patterns using qRT-PCR analysis. (a) Cdk1, (b) keratin 8, (c) keratin 18, (d) myogenin, (e) myostatin, and (f) actn3.

Cdk1 from the aging category, keratin 8 and 18 from the apoptosis category, and myogenin, myostatin, and actn3 from the muscle atrophy category were selected as representative genes for the validation of gene expression using quantitative real-time PCR analysis. The result of qRT-PCR analyses for all these selected genes was similar to the result of microarray analysis discussed above. The mRNA levels from both microarray and qRT-PCR analyses are depicted in Figure 8.

### 3.7. Commonly Expressed Genes

There were 2 common genes, namely, Rps6kb1 and gelsolin, which displayed commonly regulated expressions in the 3 categories (aging, apoptotic process, and muscle atrophy) of our interest that are known to be associated with muscle degeneration and atrophy after a rotator cuff tear. Rps6kb1 in the rotator cuff tear side was downregulated (expressed 0.48 times of the control) during the acute phase, and showed increased expression during the chronic phase (expressed 0.806 times of the control). In addition, Gelsolin in the rotator cuff tear side was also downregulated (0.40 times of the control) during the acute phase and showed increased expression during the chronic phase (expressed 1.17 times of the control).

## 4. Discussion

The major findings of this study were comprehensive analysis of genetic changes with time following a rotator cuff tear and categorization of those genes associated with 10 muscle physiology, including aging, apoptosis, atrophy, and fatty acid transport.

We regarded week 1 as the acute phase and week 4 as the chronic phase, based on our previous study using the same animal model [10]. In our previous study, we detected acute inflammatory reactions in the rotator cuff muscle with a rapid increase in inflammatory cytokines at week 1; however, the inflammatory reactions almost disappeared 4 weeks after inducing the rotator cuff tear. Conversely, fat deposition with degenerative changes in the rotator cuff muscle started increasing during week 2 and a noticeable increase was observed 4 weeks after the rotator cuff tear [10]. Based on these findings, we defined week 1 after the rotator cuff tear as the acute phase and week 4 as the chronic phase.

Among a total of 39,429 genes, the gene that showed the highest increase in expression (expressed 217.7 times higher than the control) at week 1 after the rotator cuff tear was Krt18, which plays an important role in maintaining cell structure and is a major component of the intermediate filaments of epithelial cells [30]. It is also well-known to be an indicator of the progression of chronic liver diseases because it is related to apoptosis [31]. In the present study, a rapid increase in Krt18 expression was found during the acute phase after inducing the rotator cuff tear. We considered that the natural apoptotic process in muscle cells started considerably early after the rotator cuff tear, and if the apoptotic process progressed faster than the restoration process of damaged myocytes, a permanent and irreversible damage to the rotator cuff muscle may occur and the outcome may be worse even after a successful rotator cuff repair.

In the aging category, among all genes, the expression of lgfbp2 (insulin-like growth factor binding protein 2) was the highest at week 1 after the rotator cuff tear. This suggested that the muscle damage induced by the rotator cuff tear affected the aging process of myocytes. Davalos et al. showed that the IGF-binding proteins were highly expressed in aged fibroblast cells, which supported our results [32]. Particularly, IL-6 and Ccl2, which are adhesion molecules known to be secreted from aged cells, and Vcam1 interacted with each other and showed high expression as shown in the STRING network. In the apoptosis category, Birc5 (baculoviral IAP repeat-containing 5), which is known to be a survivin like krt 18, significantly increased (expressed 55.6 times of the control) during the acute phase. This survivin is known to influence cell division and cause cell death inhibition. Hence, it is related to tissue injury and healing [33]. In addition, this gene interacts with Rps6ka2 [34], which is related to cell survival; caspase 14 [35] and Cdk1 [36], which play important roles in cell growth and apoptosis; and MELK [37], which is related to cell proliferation, apoptosis, RNA processing, and embryonic development. Birc5, which is known to play an important role in apoptosis, seemed to play a similar role in the rotator cuff muscle after the rotator cuff tear. Only 11 among the 39,429 genes analyzed belonged to the muscle atrophy category. Among these, Myog (myogenin), which is known to play an important role in the differentiation of myocytes, showed the highest expression during the acute phase after rotator cuff tear as expected. On the contrary, Mstn (myostatin), which is known to play an inhibitory role during myogenesis, exhibited the lowest expression. These results suggested that myogenin rapidly increases during the early phase after the rotator cuff tear in order to regenerate damaged muscles (with low myostatin expression) and decreases as muscle atrophy and degeneration progressed with time. It was interesting that Gatm (L-arginine: glycine amidinotransferase), which is known to be related to obesity associated with creatine metabolism [38], showed the highest expression after myogenin. In addition, two new genes, namely, Cflar and Ppargc1a, which showed reverse patterns of expression between the acute and chronic phases (Cflar: CASP8 and FADD-like apoptosis regulator, expression decreased from 2.442 times during the acute phase to 0.935 times during the chronic phase; Ppargc1a, expression changed from 0.216 times during the acute phase to 0.763 times during the chronic phase), although not significant, can be studied as novel targets associated with changes in muscle atrophy after rotator cuff tear or repair. In the fatty acid transport category, the expression of ApoE (Apolipoprotein E) was noticeable. ApoE is known to play a pivotal role in lipid homeostasis [39], and was also highly expressed among the cell proliferation category genes. This gene can be analyzed in future studies for understanding its role in the reversal of fatty infiltration after rotator cuff repair. In this study, we observed two genes (Rps6kb1 and gelsolin) which showed common expression patterns across 3 categories, namely aging, apoptosis, and muscle atrophy. Rps6kb1 is known to play an important role in anabolic signaling by increasing lipid accumulation in the adipose tissue and inducing skeletal muscle hypertrophy [40], whereas gelsolin is known to be a physiological effector of apoptotic morphological changes after being cleaved by caspase 3 [41]. In this study, low expression of Rps6kb1 during the acute phase (expressed 0.48 times of the control) and high expression during the chronic phase (expressed 0.81 times of the control) suggested muscle damage recovery over time. Conversely, low expression of gelsolin during the acute phase (expressed 0.40 times of the control) and increased expression during the chronic phase (expressed 1.17 times of the control) potentially indicates ongoing apoptotic processes in rotator cuff muscles against the force to restore the damaged muscle. These two key genes may link and control muscle degeneration and sarcopenia after the rotator cuff tear.

The strength of our study is that this is the first study which comprehensively analyzed time-dependent expression of genes belonging to different categories associated with muscle physiology after making a rotator cuff tear. This finding may help to understand the rotator cuff tear-related muscle changes and, further it may improve various detrimental outcomes of rotator cuff repair surgery by regulation of the potent key molecule or its signal pathway.

Nevertheless, this study has several limitations that require consideration. First, this study was an animal-based study. Differences in anatomic features, different injury and healing reactions, and genetic variations between humans and rats limit generalization of the results. In this study, we established rotator cuff tear (RCT) model by injuring supraspinatus to make the most similar condition to the real clinical situation because most tears occur in the supraspinatus tendon in clinical situation. However, we humbly admit that 2 tendons (supraspinatus + infraspinatus) tear model may be better to mimic the muscle degenerative changes compared to the 1 tendon model, and there could exist a likelihood of self-healing in a mouse supraspinatus tear model. Thus, we have to be cautious while interpretation of results. Second, we investigated gene expression patterns in the rotator cuff muscles up to 4 weeks after making the tear, based on the results of our previous study [10]; however, there can be more biological and genetic changes during later time points (more than 4 weeks). Finally, molecular pathways underlying changes in muscle physiology after a rotator cuff tear were not identified in this study. This should be the next step of the study.

## 5. Conclusions

(i) Rotator cuff tear induces specific genes associated with changes in muscle physiology such as aging, apoptosis, muscle atrophy, and fatty acid transport.

(ii) Several genes which are significantly altered after rotator cuff tear may play key roles in controlling muscle degeneration after a rotator cuff tear.

(iii) Mouse rotator cuff tear model could be a good approach to understand the many aspects of the molecular changes in injured muscle.

## Figures and Tables

**(a):** 

	Total	10 categories associated with muscle physiology									
1 week	Total	Aging	Angio.	Apop.	Diff.	Migr.	Prolif.	ECM	Infl.	FA tr.	Atro

Total gene number	39,429	370	443	1,100	4,701	994	788	683	532	66	19

% of Total	100	0.94	1.12	2.79	11.92	2.52	2	1.73	1.35	0.17	0.05

Up significant (n)	8,662	127	174	378	1,422	386	262	264	249	10	3

% of Up significant	22	34.3	39.3	34.4	30.2	38.8	33.2	38.7	46.8	15.2	15.8

Down significant (n)	3,516	40	30	113	436	75	45	54	31	13	9

% of Down significant	8.9	10.8	6.8	10.3	9.3	7.5	5.7	7.9	5.8	19.7	47.4

Total significant (n)	**12,178**	**184**	**203**	**525**	**1,867**	**463**	**300**	**267**	**278**	**23**	**11**

% of Total significant	30.9	45.1	46	44.6	39.5	46.4	39	46.6	52.6	34.8	63.2

**(b):** 

	Total	10 categories associated with muscle physiology									
4 weeks	Total	Aging	Angio.	Apop.	Diff.	Migr.	Prolif.	ECM	Infl.	FA tr.	Atro

Total gene number	39,429	370	443	1,100	4,701	994	788	683	532	66	19

% of Total	100	0.94	1.12	2.79	11.92	2.52	2	1.73	1.35	0.17	0.05

Up Significant (n)	1,233	21	20	52	203	59	35	31	46	2	1

% of Up Significant	3.1	5.7	4.5	4.7	4.3	5.9	4.4	4.5	8.6	3	5.3

Down Significant (n)	1,137	8	9	34	150	31	32	12	15	0	4

% of Down Significant	2.9	2.2	2	3.1	3.2	3.1	4.1	1.8	2.8	0	21.1

Total Significant (n)	**2,370**	**29**	**29**	**86**	**353**	**90**	**67**	**43**	**61**	**2**	**5**

% of Total Significant	6	7.8	6.5	7.8	7.5	9.1	8.5	6.3	11.5	3	26.3

Angio., angiogenesis; Apop., apoptosis; Diff., cell differentiation; Migr., cell migration; Prolif., cell proliferation; ECM, extracellular matrix; Infl., inflammation; FA tr., fatty acid transport; Atro., muscle atrophy.

Up significant means the genes with the fold change of rotator cuff tear/control of more than 2 the raw values (log2) of more than 4.

Down significant means the genes with the fold change of rotator cuff tear/control of less than 1/2 with the raw values (log2) of more than 4.

**(a):** 

Gene symbol	Genbank accession	Fold change	Gene name
Upregulated			

Krt18	NM_010664	217.799	keratin 18

Sirpb1b	NM_001173460	150.129	signal-regulatory protein beta 1B

Cd300lf	NM_001169153	128.186	CD300 antigen like family member F

Ms4a4c	NM_029499	122.342	membrane-spanning 4-domains, subfamily A, member 4C

Plekhh1	NM_181073	119.491	pleckstrin homology domain containing, family H (with MyTH4 domain) member 1

Cd5l	NM_009690	104.575	CD5 antigen-like

Krt8	AK166854	104.209	keratin 8

Myh8	AK081482	95.091	myosin, heavy polypeptide 8, skeletal muscle, perinatal

LOC102642410	XM_006544504	94.351	tyrosine-protein phosphatase non-receptor type substrate 1-like

Myh3	NM_001099635	92.621	myosin, heavy polypeptide 3, skeletal muscle, embryonic

Downregulated			

Pitx1	NM_011097	0.025	paired-like homeodomain transcription factor 1

Adam28	NM_183366	0.025	a disintegrin and metallopeptidase domain 28

Rnf180	NM_027934	0.027	ring finger protein 180

Gm5860	NR_040659	0.029	predicted gene 5860

H2-M9	NM_008205	0.03	histocompatibility 2, M region locus 9

Smco1	NM_183283	0.03	single-pass membrane protein with coiled-coil domains 1

AU042410	BB209673	0.031	expressed sequence AU042410

Kcnc1	NM_001112739	0.031	potassium voltage gated channel, Shaw-related subfamily, member 1

8430426J06Rik	NR_077229	0.032	RIKEN cDNA 8430426J06 gene

Gm10822	AK144860	0.033	predicted gene 10822

**(b):** 

Gene symbol	Genbank accession	Fold change	Gene name
Upregulated			

Gm7325	NM_001177470	45.121	predicted gene 7325

Plekhh1	NM_181073	42.051	pleckstrin homology domain containing, family H (with MyTH4 domain) member 1

Ces2b	NM_198171	28.227	carboxylesterase 2B

Ces2c	NM_145603	25.457	carboxylesterase 2C

Sln	NM_025540	24.155	sarcolipin

Krt18	NM_010664	22.114	keratin 18

Myh3	NM_001099635	17.984	myosin, heavy polypeptide 3, skeletal muscle, embryonic

Krt8	AK166854	16.550	keratin 8

Zfp735	NM_001126489	15.836	zinc finger protein 735

Epha1	NM_023580	15.534	Eph receptor A1

Downregulated			

Dok6	NM_001039173	0.063	docking protein 6

Snhg11	NM_175692	0.072	small nucleolar RNA host gene 11

Olfr981	NM_146286	0.087	olfactory receptor 981

Pitx1	NM_011097	0.087	paired-like homeodomain transcription factor 1

Gm12	NM_001195544	0.093	predicted gene 12

Olfr586	NM_147111	0.098	olfactory receptor 586

Samd11	XR_881429	0.101	sterile alpha motif domain containing 11

Dnase1	NM_010061	0.111	deoxyribonuclease I

1190003K10Rik	NM_001195435	0.114	RIKEN cDNA 1190003K10 gene

Gpr176	NM_201367	0.117	G protein-coupled receptor 176

**(a):** 

Gene symbol	Genbank accession	Fold change	Gene name
Upregulated			

Igfbp2	NM_008342	55.685	insulin-like growth factor binding protein 2

Arg1	NM_007482	54.973	arginase, liver

Cdk1	NM_007659	34.242	cyclin-dependent kinase 1

Cdkn2a	NM_009877	30.112	cyclin-dependent kinase inhibitor 2A

Aldh3a1	NM_007436	19.548	aldehyde dehydrogenase family 3, subfamily A1

Ddc	NM_001190448	17.423	dopa decarboxylase

Ccl2	NM_011333	16.814	chemokine (C-C motif) ligand 2

Inpp5d	NM_010566	15.961	inositol polyphosphate-5-phosphatase D

Mpo	NM_010824	14.836	myeloperoxidase

Rad54l	NM_009015	13.26	RAD54 like (*S. cerevisiae*)

Downregulated			

Tfcp2l1	NM_023755	0.125	transcription factor CP2-like 1

Nat1	NM_008673	0.146	N-acetyl transferase 1

Ppargc1a	NM_008904	0.153	peroxisome proliferative activated receptor, gamma, coactivator 1 alpha

Atp8a2	NM_015803	0.157	ATPase, aminophospholipid transporter-like, class I, type 8A, member 2

Pde4d	NM_011056	0.183	phosphodiesterase 4D, cAMP specific

Endog	NM_007931	0.195	endonuclease G

Cyp1a1	NM_009992	0.199	cytochrome P450, family 1, subfamily a, polypeptide 1

Kl	NM_013823	0.2	klotho

Cryab	NM_001289782	0.21	crystallin, alpha B

P2ry1	NM_008772	0.216	purinergic receptor P2Y, G-protein coupled 1

**(b):** 

Gene symbol	Genbank accession	Fold change	Gene name
Upregulated			

Slc1a2	NM_001077514	10.851	solute carrier family 1 (glial high affinity glutamate transporter), member 2

Krtap4-16	NM_001013823	9.991	keratin associated protein 4-16

Ghrhr	NM_001003685	9.285	growth hormone releasing hormone receptor

Ddc	NM_001190448	8.068	dopa decarboxylase

Aldh3a1	NM_007436	7.897	aldehyde dehydrogenase family 3, subfamily A1

Cdkn2a	NM_009877	6.885	cyclin-dependent kinase inhibitor 2A

Igfbp2	NM_008342	5.164	insulin-like growth factor binding protein 2

Retn	NM_001204959	3.729	resistin

Adra1a	NM_013461	3.523	adrenergic receptor, alpha 1a

Cnr1	NM_007726	3.51	cannabinoid receptor 1 (brain)

Downregulated			

Il10	NM_010548	0.195	interleukin 10

Cdk1	NM_007659	0.227	cyclin-dependent kinase 1

Glrx2	NM_001038592	0.325	glutaredoxin 2 (thioltransferase)

Tfcp2l1	NM_023755	0.391	transcription factor CP2-like 1

Fos	NM_010234	0.438	FBJ osteosarcoma oncogene

Atp8a2	NM_015803	0.459	ATPase, aminophospholipid transporter-like, class I, type 8A, member 2

Hmga1	NM_001166546	0.462	high mobility group AT-hook 1

Bcl2	NM_177410	0.464	B-cell leukemia/lymphoma 2

Srf	NM_020493	0.482	serum response factor

Arntl	NM_007489	0.519	aryl hydrocarbon receptor nuclear translocator-like

**(a):** 

Gene symbol	Genbank accession	Fold change	Gene name
Upregulated			

Krt18	NM_010664	217.799	keratin 18

Cd5l	NM_009690	104.575	CD5 antigen-like

Krt8	AK166854	104.209	keratin 8

Serpina3g	NM_009251	85.473	serine (or cysteine) peptidase inhibitor, clade A, member 3G

Melk	NM_010790	72.784	maternal embryonic leucine zipper kinase

Birc5	NM_009689	55.603	baculoviral IAP repeat-containing 5

Gpr65	NM_008152	54.323	G-protein coupled receptor 65

Gzma	NM_010370	52.943	granzyme A

Top2a	NM_011623	44.234	topoisomerase (DNA) II alpha

Bcl2a1d	NM_007536	43.608	B-cell leukemia/lymphoma 2-related protein A1d

Downregulated			

Dnase1	NM_010061	0.051	deoxyribonuclease I

Casp14	NM_009809	0.099	caspase 14

Robo2	BC055333	0.104	roundabout homolog 2 (*Drosophila*)

Camk2a	NM_009792	0.107	calcium/calmodulin-dependent protein kinase II alpha

Il24	NM_053095	0.112	interleukin 24

Chac1	NM_026929	0.119	ChaC, cation transport regulator 1

Nr4a1	NM_010444	0.122	nuclear receptor subfamily 4, group A, member 1

Mtfp1	NM_026443	0.124	mitochondrial fission process 1

Rps6ka2	NM_011299	0.156	ribosomal protein S6 kinase, polypeptide 2

Casr	NM_013803	0.158	calcium-sensing receptor

**(b):** 

Gene symbol	Genbank accession	Fold change	Gene name
Upregulated			

Krt18	NM_010664	22.114	keratin 18

Krt8	AK166854	16.55	keratin 8

Bcl2l14	NM_025778	14.994	BCL2-like 14 (apoptosis facilitator)

Tox3	NM_172913	10.794	TOX high mobility group box family member 3

Fasl	NM_010177	10.487	Fas ligand (TNF superfamily, member 6)

Pak7	NM_172858	9.86	p21 protein (Cdc42/Rac)-activated kinase 7

Fgf21	NM_020013	7.87	fibroblast growth factor 21

Avp	NM_009732	7.557	arginine vasopressin

Nlrc4	NM_001033367	7.471	NLR family, CARD domain containing 4

Cdkn2a	NM_009877	6.885	cyclin-dependent kinase inhibitor 2A

Downregulated			

Dnase1	NM_010061	0.111	deoxyribonuclease I

Cd27	NM_001033126	0.117	CD27 antigen

Fgf8	NM_010205	0.162	fibroblast growth factor 8

AY074887	NM_145229	0.168	cDNA sequence AY074887

Tnf	NM_013693	0.205	tumor necrosis factor

Naip1	NM_008670	0.217	NLR family, apoptosis inhibitory protein 1

Cdk1	NM_007659	0.227	cyclin-dependent kinase 1

Epha7	NM_010141	0.239	Eph receptor A7

Tiam1	NM_009384	0.253	T-cell lymphoma invasion and metastasis 1

Bub1	NM_009772	0.284	budding uninhibited by benzimidazoles 1 homolog (*S. cerevisiae*)

**(a):** 

Gene symbol	Genbank accession	Fold change	Gene name
Upregulated			

Myog	NM_031189	38.535	myogenin

Gatm	NM_025961	8.862	glycine amidinotransferase (L-arginine:glycine amidinotransferase):energy metabolism of muscle

Cflar	NM_009805	2.442	CASP8 and FADD-like apoptosis regulator

Rps6kb1	NM_028259	1.287	ribosomal protein S6 kinase, polypeptide 1

Downregulated			

Il15	NM_008357	0.727	interleukin 15

Tbce	NM_178337	0.648	tubulin-specific chaperone E

Gsn	NM_146120	0.439	Gelsolin:actin-binding protein

Trim63	NM_001039048	0.435	tripartite motif-containing 63

Actn3	NM_013456	0.279	actinin alpha 3:alpha-actinin skeletal muscle isoform 3

Ppargc1a	NM_008904	0.216	peroxisome proliferative activated receptor, gamma, coactivator 1 alpha

Mstn	NM_010834	0.108	myostatin

**(b):** 

Gene symbol	Genbank accession	Fold change	Gene name
Upregulated			

Myog	NM_031189	11.945	myogenin

Gatm	NM_025961	1.712	glycine amidinotransferase (L-arginine:glycine amidinotransferase)

Gsn	NM_146120	1.249	gelsolin

Rps6kb1	NM_028259	1.245	ribosomal protein S6 kinase, polypeptide 1

Downregulated			

Il15	NM_008357	0.98	interleukin 15

Cflar	NM_207653	0.935	CASP8 and FADD-like apoptosis regulator

Ppargc1a	NM_008904	0.763	peroxisome proliferative activated receptor, gamma, coactivator 1 alpha

Tbce	NM_178337	0.712	tubulin-specific chaperone E

Actn3	NM_013456	0.494	actinin alpha 3

Trim63	NM_001039048	0.387	tripartite motif-containing 63

Mstn	NM_010834	0.378	myostatin

**(a):** 

Gene symbol	Genbank accession	Fold change	Gene name
Upregulated			

Mfsd2a	NM_029662	12.606	major facilitator superfamily domain containing 2A

Apoe	NM_009696	12.229	apolipoprotein E

Drd4	NM_007878	10.607	dopamine receptor D4

Slc27a6	NM_001081072	7.557	solute carrier family 27 (fatty acid transporter), member 6

Pla2g4a	NM_008869	6.457	phospholipase A2, group IVA (cytosolic, calcium-dependent)

Anxa1	NM_010730	5.517	annexin A1

Plin2	NM_007408	5.018	perilipin 2

Pla2g2e	NM_012044	3.407	phospholipase A2, group IIE

Pla2g1b	NM_011107	3.384	phospholipase A2, group IB, pancreas

Hnf1a	M57966	2.029	HNF1 homeobox A

Downregulated			

Fabp3	NM_010174	0.131	fatty acid binding protein 3, muscle and heart

Pla2g5	NM_001122954	0.15	phospholipase A2, group V

Cpt1b	NM_009948	0.186	carnitine palmitoyltransferase 1b, muscle

Pla2g12a	NM_183423	0.262	phospholipase A2, group XIIA

Got2	NM_010325	0.289	glutamic-oxaloacetic transaminase 2, mitochondrial

Slc27a2	NM_011978	0.299	solute carrier family 27 (fatty acid transporter), member 2

Slc27a1	NM_011977	0.32	solute carrier family 27 (fatty acid transporter), member 1

Acsl1	NM_007981	0.354	acyl-CoA synthetase long-chain family member 1

Pnpla8	NM_026164	0.372	patatin-like phospholipase domain containing 8

Pla2g2c	NM_008868	0.408	phospholipase A2, group IIC

**(b):** 

Gene symbol	Genbank accession	Fold change	Gene name
Upregulated			

Nmur2	NM_153079	3.742	neuromedin U receptor 2

Apoe	NM_009696	2.729	apolipoprotein E

Pla2g2d	NM_011109	2.704	phospholipase A2, group IID

Pparg	NM_011146	1.56	peroxisome proliferator activated receptor gamma

Proca1	XM_006532963	1.548	protein interacting with cyclin A1

Pla2g2e	NM_012044	1.539	phospholipase A2, group IIE

Pla2g4a	NM_008869	1.485	phospholipase A2, group IVA (cytosolic, calcium-dependent)

Anxa1	NM_010730	1.377	annexin A1

Pla2g2a	NM_001082531	1.286	phospholipase A2, group IIA (platelets, synovial fluid)

Mfsd2a	NM_029662	1.268	major facilitator superfamily domain containing 2A

Downregulated			

Pla2g1b	NM_011107	0.428	phospholipase A2, group IB, pancreas

Slc27a5	NM_009512	0.456	solute carrier family 27 (fatty acid transporter), member 5

Pla2g3	NM_172791	0.511	phospholipase A2, group III

Hnf1a	M57966	0.529	HNF1 homeobox A

Fabp3	NM_010174	0.587	fatty acid-binding protein 3, muscle and heart

Pla2g2f	NM_012045	0.592	phospholipase A2, group IIF

Abcc4	NM_001033336	0.592	ATP-binding cassette, sub-family C (CFTR/MRP), member 4

Pnpla8	NM_026164	0.614	patatin-like phospholipase domain containing 8

Ppard	NM_011145	0.628	peroxisome proliferator activator receptor delta

Pla2g5	NM_001122954	0.661	phospholipase A2, group V

## Data Availability

The data used to support the findings of this study are available from the corresponding author upon request.

## Supplementary Materials

Supplementary Materials “Supplementary Figures” and “Supplementary Table” show gene expression patterns and the top 10 up- and downregulated genes in an additional muscle physiology-related category after rotator cuff tear, respectively.
