# Supplementary material for: Gene Expression Patterns Analysis in the Supraspinatus Muscle after a Rotator Cuff Tear in a Mouse Model
Source: Biomed Res Int. 2018 Dec 23;2018:5859013. doi: 10.1155/2018/5859013 (PMC6323466; doi:10.1155/2018/5859013)
Supplement: Supplementary Materials — “Supplementary Figures” and “Supplementary Table” show gene expression patterns and the top 10 up- and downregulated genes in an additional muscle physiology-related category after rotator cuff tear, respectively. [file 5859013.f1.pdf]

Supplementary Figure 1

a)

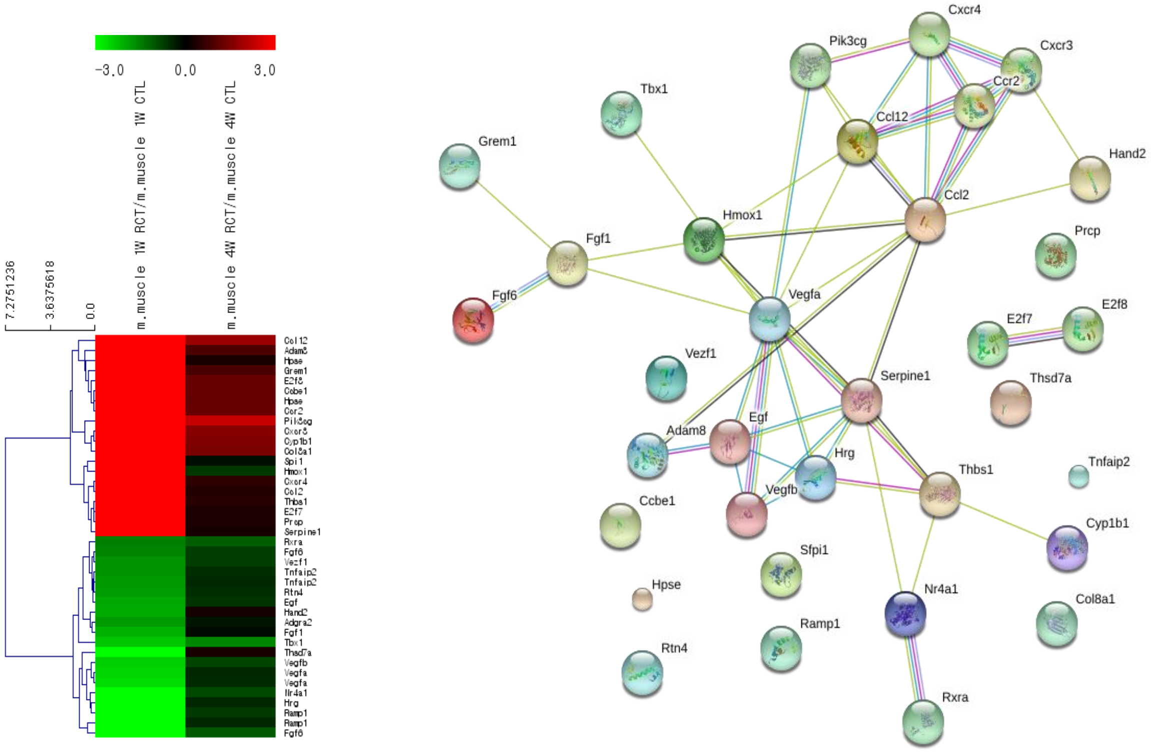

b)

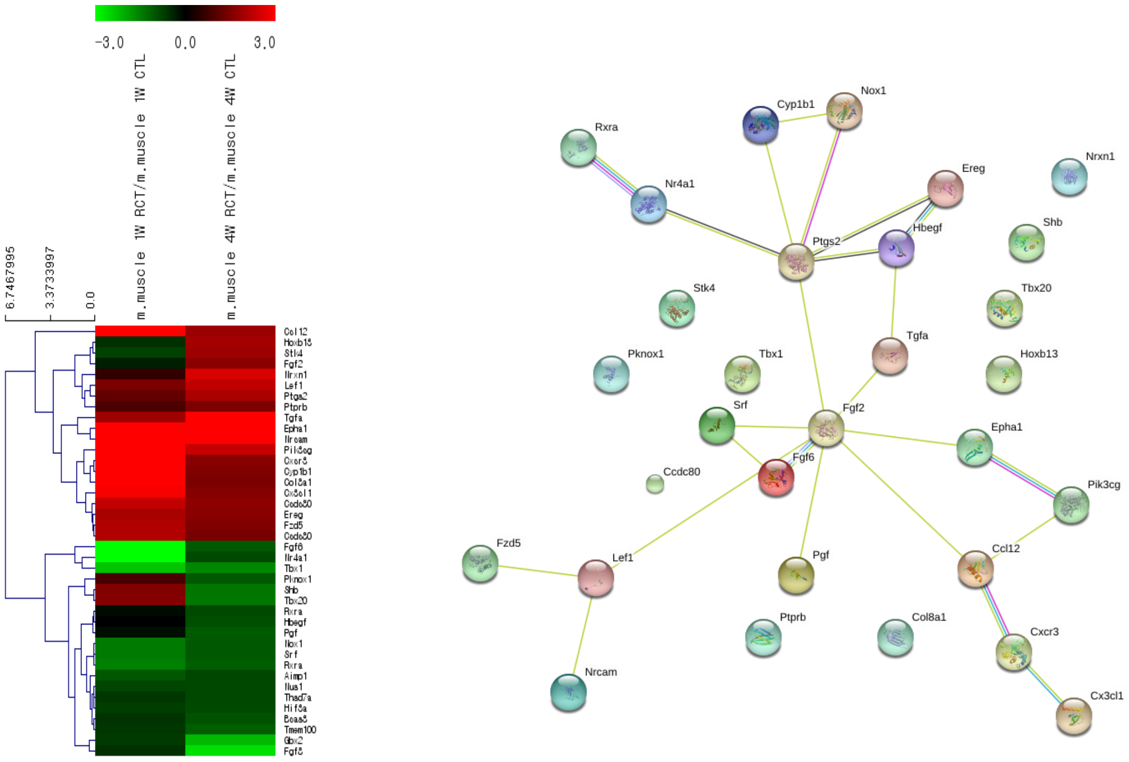

Supplementary Figure 2

a)

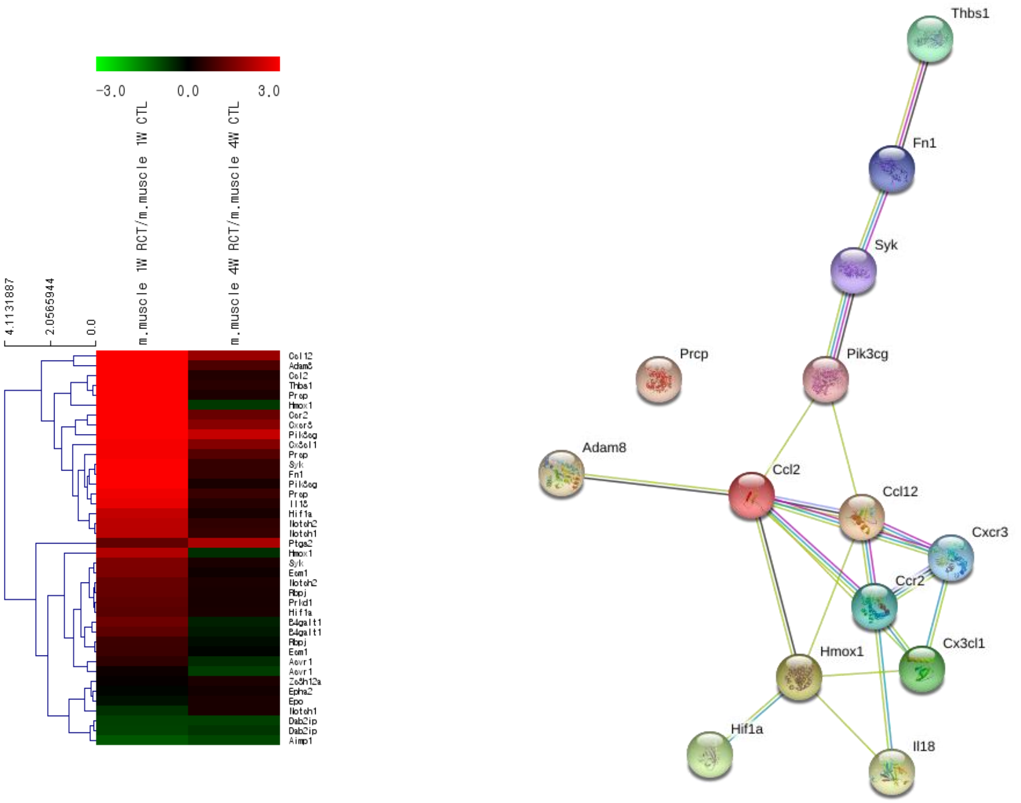

b)

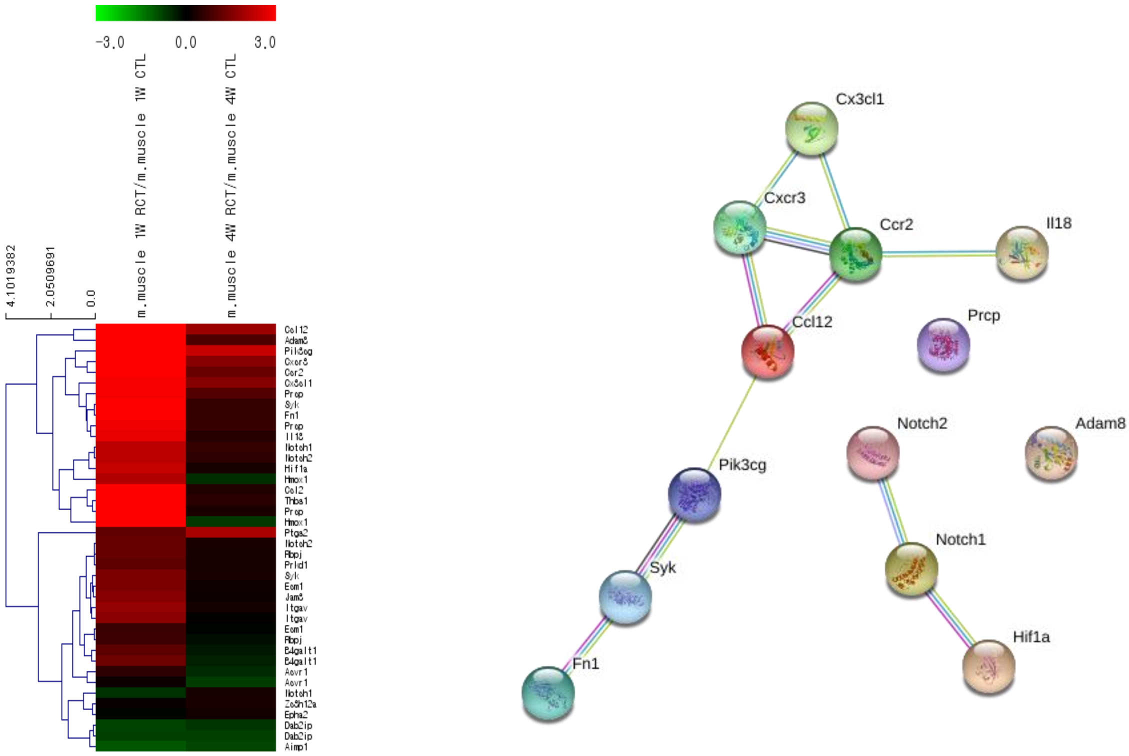

Supplementary Figure 3

a)

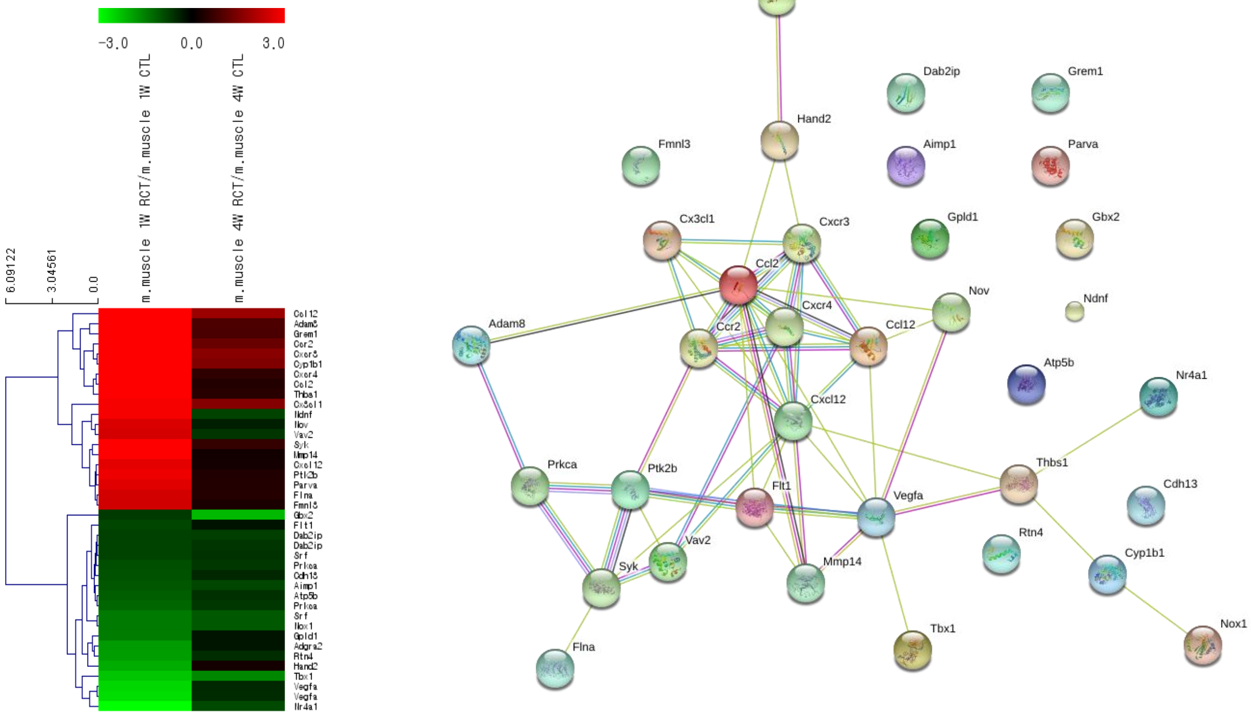

b)

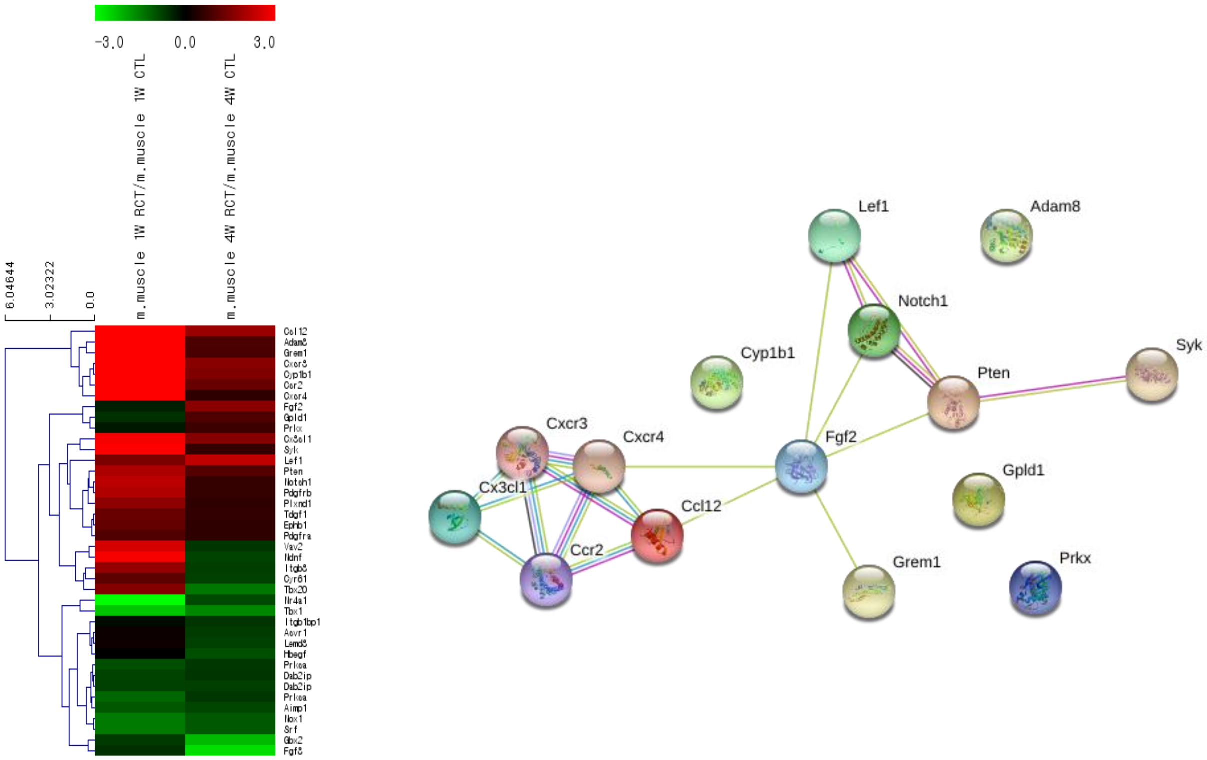

Supplementary Figure 4

a)

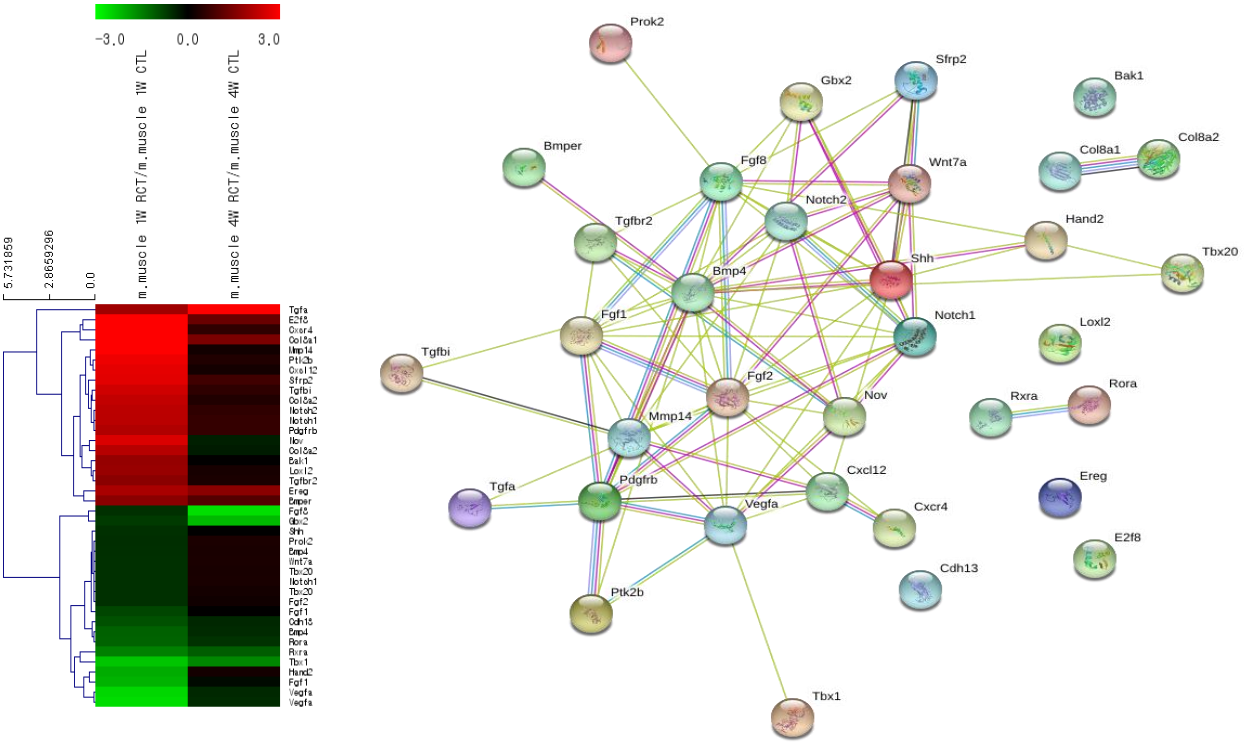

b)

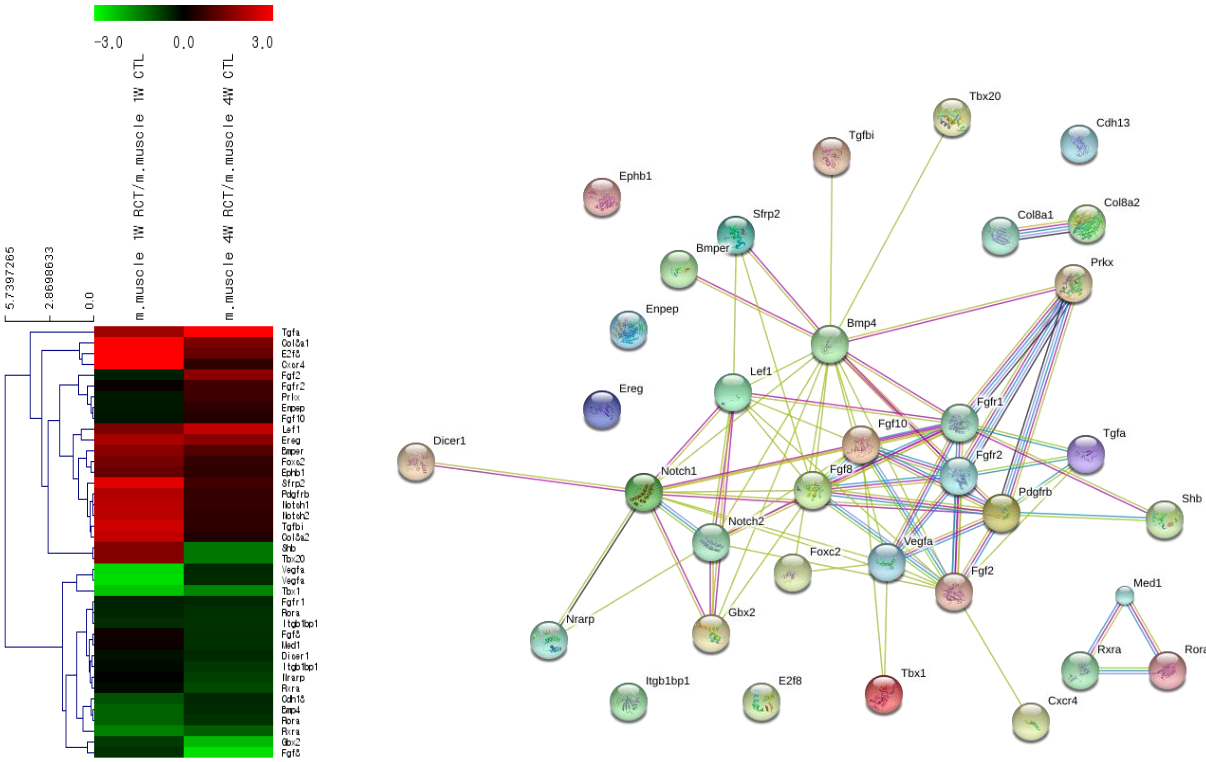

Supplementary Figure 5

a)

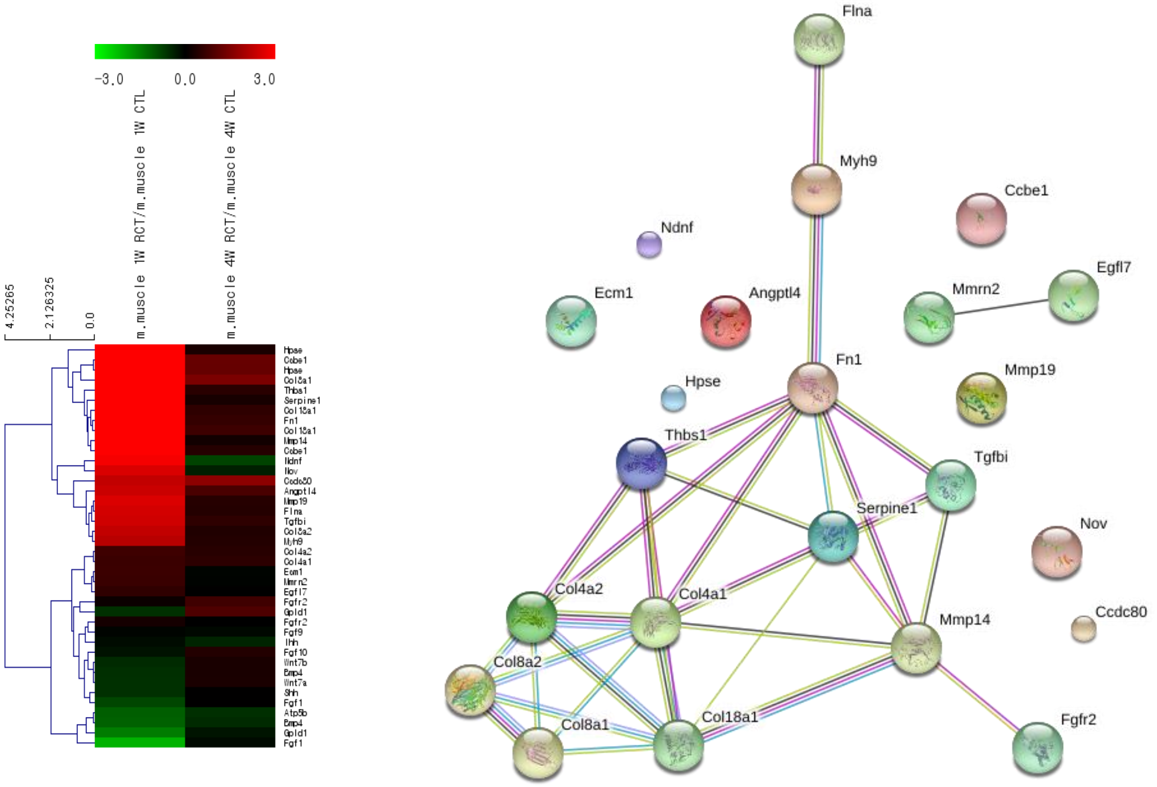

b)

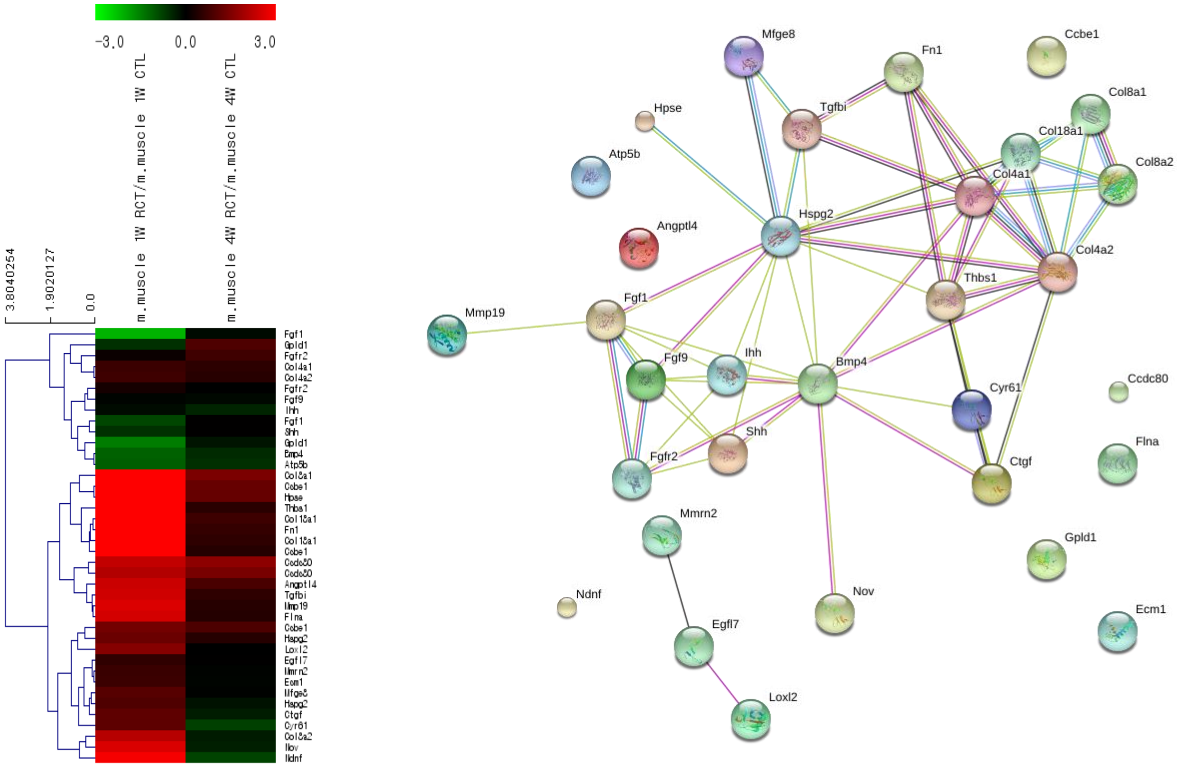

Supplementary Figure 6

a)

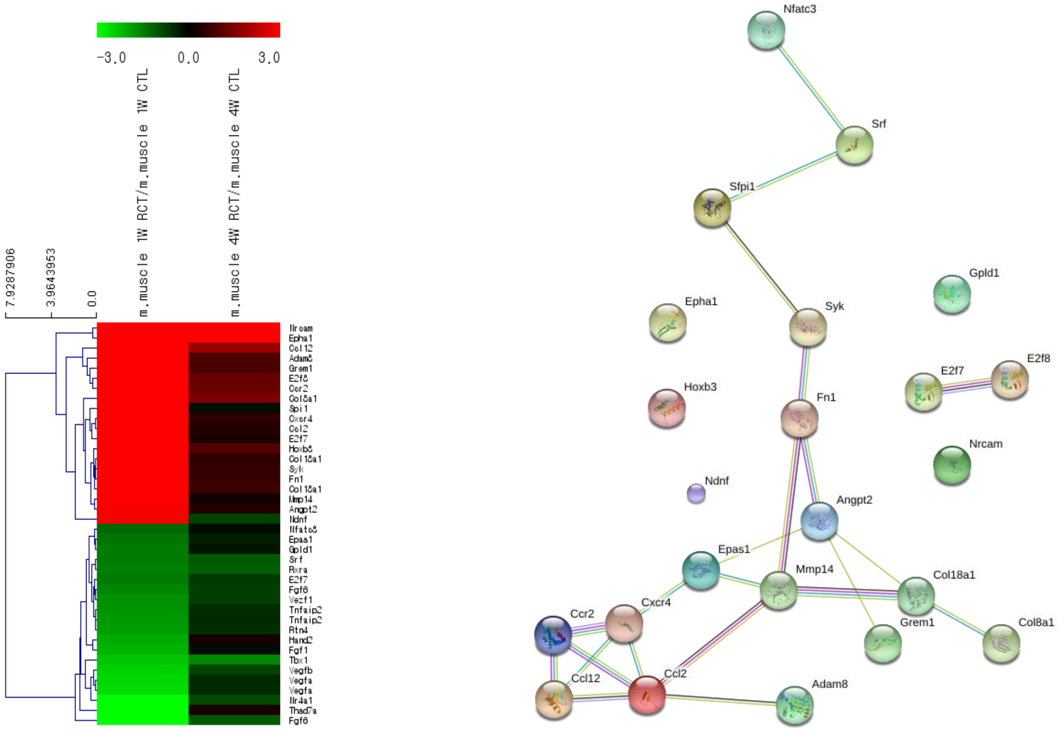

b)

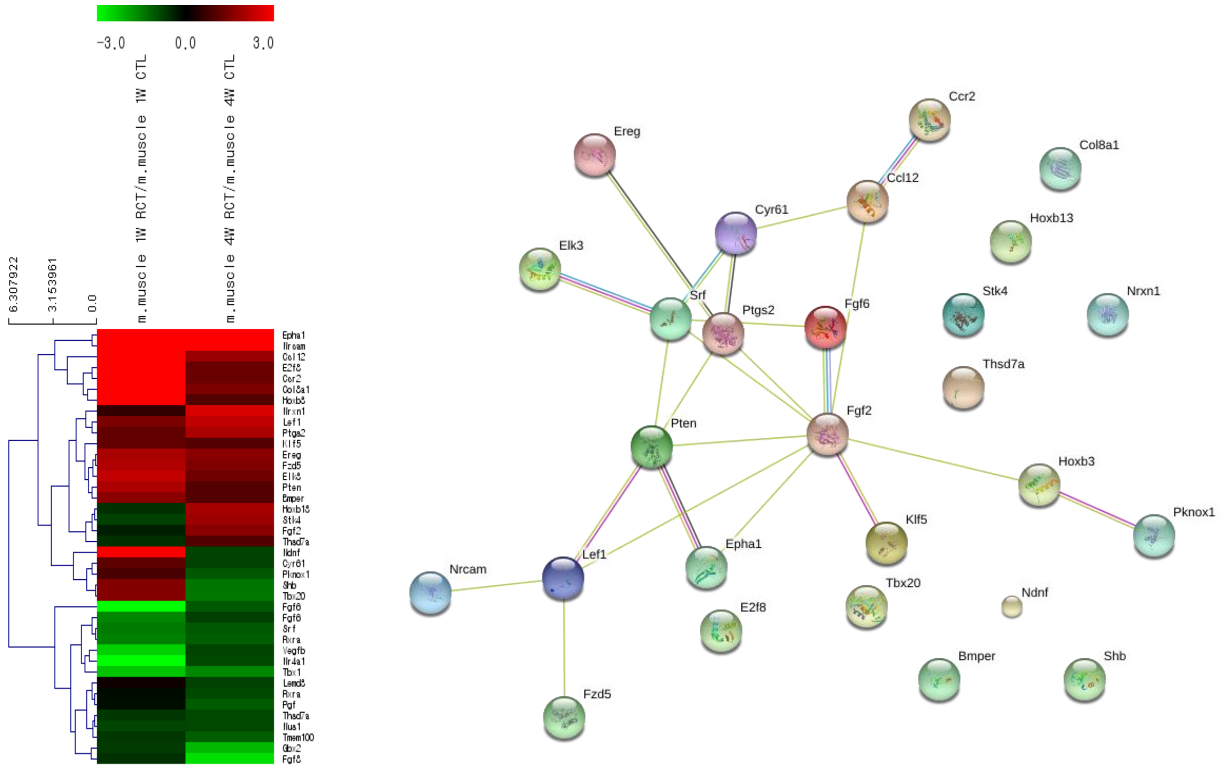

Supplementary Figure Legends

Supplementary Figure 1. Differential expression patterns in hierarchical clustering and the STRING network for the angiogenesis category (a, week 1 after tear; b, week 4 after tear). Left side shows differential expression patterns and hierarchical clustering, and right side shows the STRING network for the angiogenesis category.

Supplementary Figure 2. Differential expression patterns in hierarchical clustering and the STRING network for the inflammation category (a, week 1 after tear; b, week 4 after tear).

Supplementary Figure 3. Differential expression patterns in hierarchical clustering and the STRING network for the cell migration category (a, week 1 after tear; b, week 4 after tear).

Supplementary Figure 4. Differential expression patterns in hierarchical clustering and the STRING network for the cell proliferation category (a, week 1 after tear; b, week 4 after tear).

Supplementary Figure 5. Differential expression patterns in hierarchical clustering and the STRING network for the extracellular matrix category (a, week 1 after tear; b, week 4 after tear).

Supplementary Figure 6. Differential expression patterns in hierarchical clustering and

the STRING network for the cell differentiation category (a, week 1 after tear; b, week 4 after tear).

Supplementary Table I. Gene expression patterns in the angiogenesis category after rotator cuff tear (a, week 1 after tear; b, week 4 after tear)

a)

Gene symbol	Genbank accession	Fold change	Gene name
Upregulated			
Ccl12	NM_011331	59.221	chemokine (C-C motif) ligand 12
Adam8	NM_007403	42.215	a disintegrin and metallopeptidase domain 8
Grem1	NM_011824	27.727	gremlin 1
Hpse	NM_152803	26.165	heparanase
E2f8	NM_001013368	23.223	E2F transcription factor 8
Ccbe1	NM_178793	19.573	collagen and calcium binding EGF domains 1
Cxcr4	NM_009911	17.723	chemokine (C-X-C motif) receptor 4
Ccr2	NM_009915	17.505	chemokine (C-C motif) receptor 2
Ccl2	NM_011333	16.814	chemokine (C-C motif) ligand 2
Cxcr3	NM_009910	16.097	chemokine (C-X-C motif) receptor 3
Downregulated			
Fgf6	NM_010204	0.055	fibroblast growth factor 6
Ramp1	NM_016894	0.068	receptor (calcitonin) activity modifying protein 1
Hrg	NM_053176	0.11	histidine-rich glycoprotein
Thsd7a	NM_001164805	0.116	thrombospondin, type I, domain containing 7A
Nr4a1	NM_010444	0.122	nuclear receptor subfamily 4, group A, member 1
Vegfa	NM_001025250	0.164	vascular endothelial growth factor A
Vegfb	NM_011697	0.183	vascular endothelial growth factor B
Tbx1	NM_001285472	0.2	T-box 1
Fgf1	NM_010197	0.231	fibroblast growth factor 1
Hand2	NM_010402	0.244	heart and neural crest derivatives expressed transcript 2

b)

Gene symbol	Genbank accession	Fold change	Gene name
Upregulated			
Tgfa	NM_031199	19.619	transforming growth factor alpha
Epha1	NM_023580	15.534	Eph receptor A1
Nrcam	NM_176930	12.289	neuronal cell adhesion molecule
Nrxn1	NM_020252	5.95	neurexin I
Pik3cg	NM_020272	5.095	phosphoinositide-3-kinase, catalytic, gamma polypeptide
Lef1	NM_010703	4.837	lymphoid enhancer binding factor 1
Ptgs2	NM_011198	4.047	prostaglandin-endoperoxide synthase 2
Hoxb13	NM_008267	3.856	homeobox B13
Stk4	AK030098	3.641	serine/threonine kinase 4
Ccl12	NM_011331	3.549	chemokine (C-C motif) ligand 12
Downregulated			
Fgf8	NM_010205	0.162	fibroblast growth factor 8
Gbx2	NM_010262	0.22	gastrulation brain homeobox 2
Tbx1	NM_001285472	0.33	T-box 1
Tbx20	NM_001205085	0.373	T-box 20
Shb	NM_001033306	0.384	src homology 2 domain-containing transforming protein B
Tmem100	NM_026433	0.462	transmembrane protein 100
Rxra	NM_011305	0.466	retinoid X receptor alpha
Pgf	NM_008827	0.479	placental growth factor
Pknox1	NM_016670	0.481	Pbx/knotted 1 homeobox
Fgf6	NM_010204	0.481	fibroblast growth factor 6

Supplementary Table II. Gene expression patterns in the inflammation category after rotator cuff tear (a, week 1 after tear; b, week 4 after tear)

a)

Gene symbol	Genbank accession	Fold change	Gene name
Upregulated			
Tac4	NM_053093	75.566	tachykinin 4
Mefv	NM_019453	69.174	Mediterranean fever
Ccl3	NM_011337	60.502	chemokine (C-C motif) ligand 3
Ccl12	NM_011331	59.221	chemokine (C-C motif) ligand 12
Slamf8	NM_029084	57.831	SLAM family member 8
Saa3	NM_011315	56.682	serum amyloid A 3
Ccl5	NM_013653	55.091	chemokine (C-C motif) ligand 5
Cxcl5	NM_009141	52.583	chemokine (C-X-C motif) ligand 5
Ccl8	NM_021443	48.163	chemokine (C-C motif) ligand 8
Cxcl10	NM_021274	47.254	chemokine (C-X-C motif) ligand 10
Downregulated			
Nlrp4e	NM_001004194	0.128	NLR family, pyrin domain containing 4E
Il20	NM_021380	0.131	interleukin 20
Klk1b1	NM_010645	0.158	kallikrein 1-related peptidase b1
Itgb6	NM_021359	0.168	integrin beta 6
Cd27	NM_001033126	0.192	CD27 antigen
Ccl24	NM_019577	0.233	chemokine (C-C motif) ligand 24
Il17re	NM_001034031	0.253	interleukin 17 receptor E
Hmgb1	NM_010439	0.26	high mobility group box 1
Ccl25	NR_033527	0.277	chemokine (C-C motif) ligand 25
Tpsb2	NM_010781	0.279	tryptase beta 2

b)

Gene symbol	Genbank accession	Fold change	Gene name
Upregulated			
Ephb6	NM_001146351	10.315	Eph receptor B6
Zap70	NM_001289765	10.238	zeta-chain (TCR) associated protein kinase
Saa3	NM_011315	8.495	serum amyloid A 3
Nlrc4	NM_001033367	7.471	NLR family, CARD domain containing 4
Serpina1a	NM_001252569	7.423	serine (or cysteine) peptidase inhibitor, clade A, member 1A
Slamf8	NM_029084	6.867	SLAM family member 8
Ffar2	NM_146187	6.302	free fatty acid receptor 2
Tlr1	NM_030682	5.662	toll-like receptor 1
Ccl4	NM_013652	5.567	chemokine (C-C motif) ligand 4
Vnn1	NM_011704	5.538	vanin 1
Downregulated			
Cd27	NM_001033126	0.117	CD27 antigen
Tlr8	NM_133212	0.181	toll-like receptor 8
Il10	NM_010548	0.195	interleukin 10
Tnf	NM_013693	0.205	tumor necrosis factor
Pik3cd	NM_001164051	0.22	phosphatidylinositol 3-kinase catalytic delta polypeptide
Itgb6	NM_021359	0.251	integrin beta 6
Elf3	NM_007921	0.262	E74-like factor 3
Nlrp4e	NM_001004194	0.342	NLR family, pyrin domain containing 4E
Cela1	NM_033612	0.347	chymotrypsin-like elastase family, member 1
Tnfrsf11b	NM_008764	0.35	tumor necrosis factor receptor superfamily, member 11b (osteoprotegerin)

Supplementary Table III. Gene expression patterns in the cell migration category after rotator cuff tear (a, week 1 after tear; b, week 4 after tear)

a)

Gene symbol	Genbank accession	Fold change	Gene name
Upregulated			
Ccl4	NM_013652	88.894	chemokine (C-C motif) ligand 4
Ccl3	NM_011337	60.502	chemokine (C-C motif) ligand 3
Ccl12	NM_011331	59.221	chemokine (C-C motif) ligand 12
Slamf8	NM_029084	57.831	SLAM family member 8
Saa3	NM_011315	56.682	serum amyloid A 3
Ccl5	NM_013653	55.091	chemokine (C-C motif) ligand 5
Cxcl5	NM_009141	52.583	chemokine (C-X-C motif) ligand 5
Ccl8	NM_021443	48.163	chemokine (C-C motif) ligand 8
Cxcl10	NM_021274	47.254	chemokine (C-X-C motif) ligand 10
Ccr7	NM_007719	42.562	chemokine (C-C motif) receptor 7
Downregulated			
Foxb1	NM_022378	0.01	forkhead box B1
Isl1	NM_021459	0.06	ISL1 transcription factor, LIM/homeodomain
Nrg3	NM_008734	0.076	neuregulin 3
Ptp4a3	NM_008975	0.076	protein tyrosine phosphatase 4a3
Schip1	NM_013928	0.109	schwannomin interacting protein 1
Nlrp12	NM_001033431	0.116	NLR family, pyrin domain containing 12
Nr4a1	NM_010444	0.122	nuclear receptor subfamily 4, group A, member 1
Ntrk2	NM_001025074	0.133	neurotrophic tyrosine kinase, receptor, type 2
Fscn2	NM_172802	0.144	fascin homolog 2, actin-bundling protein, retinal (Strongylocentrotus purpuratus)
Slc37a4	NM_008063	0.149	solute carrier family 37 (glucose-6-phosphate transporter), member 4

b)

Gene symbol	Genbank accession	Fold change	Gene name
Upregulated			
Ccdc141	NM_001025576	14.524	coiled-coil domain containing 141
Nrg1	NM_178591	13.811	neuregulin 1
Dbh	NM_138942	12.413	dopamine beta hydroxylase
Gpr15	NM_001162955	10.81	G protein-coupled receptor 15
Cxcl10	NM_021274	10.808	chemokine (C-X-C motif) ligand 10
Chst4	NM_011998	10.372	carbohydrate (chondroitin 6/keratan) sulfotransferase 4
Fktn	NM_139309	9.283	fukutin
Ccl28	NM_020279	8.712	chemokine (C-C motif) ligand 28
Saa3	NM_011315	8.495	serum amyloid A 3
Mkl2	NM_181860	7.659	MKL/myocardin-like 2
Downregulated			
Cdc42bpa	NM_001033285	0.013	CDC42 binding protein kinase alpha
Abi2	NM_001198571	0.16	abl-interactor 2
Fgf8	NM_010205	0.162	fibroblast growth factor 8
Lrp8	NM_001080926	0.169	low density lipoprotein receptor-related protein 8, apolipoprotein e receptor
Ccdc141	NM_001025576	0.185	coiled-coil domain containing 141
Tnf	NM_013693	0.205	tumor necrosis factor
Gbx2	NM_010262	0.22	gastrulation brain homeobox 2
Nkx6-1	NM_144955	0.239	NK6 homeobox 1
Fam83d	NM_027975	0.247	family with sequence similarity 83, member D
Fat2	NM_001029988	0.251	FAT tumor suppressor homolog 2 (*Drosophila*)

Supplementary Table IV. Gene expression patterns in the cell proliferation category after rotator cuff tear (a, week 1 after tear; b, week 4 after tear)

a)

Gene symbol	Genbank accession	Fold change	Gene name
Upregulated			
Melk	NM_010790	72.784	maternal embryonic leucine zipper kinase
Fcgr4	NM_144559	56.317	Fc receptor, IgG, low affinity IV
Sox11	NM_009234	50.852	SRY (sex determining region Y)-box 11
Itgax	NM_021334	38.054	integrin alpha X
Bub1	NM_009772	37.56	budding uninhibited by benzimidazoles 1 homolog (*S. cerevisiae*)
Cdk1	NM_007659	34.242	cyclin-dependent kinase 1
Aspm	NM_009791	32.536	asp (abnormal spindle)-like, microcephaly associated (*Drosophila*)
Il7r	NM_008372	31.072	interleukin 7 receptor
Mcm10	NM_027290	24.968	minichromosome maintenance deficient 10 (S. cerevisiae)
Mki67	NM_001081117	23.66	antigen identified by monoclonal antibody Ki 67
Downregulated			
Sall4	NM_175303	0.083	sal-like 4 (*Drosophila*)
Wnt4	NM_009523	0.114	wingless-type MMTV integration site family, member 4
Satb1	NM_009122	0.139	special AT-rich sequence binding protein 1
Usp13	NM_001013024	0.149	ubiquitin specific peptidase 13 (isopeptidase T-3)
Hnf1b	NM_009330	0.159	HNF1 homeobox B
Vegfa	NM_001025250	0.164	vascular endothelial growth factor A
Crip3	NM_053250	0.177	cysteine-rich protein 3
Map7	NM_008635	0.186	microtubule-associated protein 7
Usp13	NM_001013024	0.188	ubiquitin specific peptidase 13 (isopeptidase T-3)
Cd27	NM_001033126	0.192	CD27 antigen

b)

Gene symbol	Genbank accession	Fold change	Gene name
Upregulated			
Tgfa	NM_031199	19.619	transforming growth factor alpha
Ephb6	NM_001146351	10.315	Eph receptor B6
Rag2	NM_009020	10.204	recombination activating gene 2
Tenm4	NM_011858	8.148	teneurin transmembrane protein 4
Sox11	NM_009234	8.056	SRY (sex determining region Y)-box 11
Rasgrf1	NM_011245	8.003	RAS protein-specific guanine nucleotide-releasing factor 1
Il7r	NM_008372	7.643	interleukin 7 receptor
Ifna5	NM_010505	7.339	interferon alpha 5
Slamf6	NM_030710	7.185	SLAM family member 6
Neurod4	NM_007501	6.992	neurogenic differentiation 4
Downregulated			
Dct	NM_010024	0.108	dopachrome tautomerase
Cd27	NM_001033126	0.117	CD27 antigen
Fgf8	NM_010205	0.162	fibroblast growth factor 8
Sox5	NM_011444	0.182	SRY (sex determining region Y)-box 5
Sall4	NM_175303	0.200	sal-like 4 (*Drosophila*)
Tnf	NM_013693	0.205	tumor necrosis factor
Gbx2	NM_010262	0.22	gastrulation brain homeobox 2
Cdk1	NM_007659	0.227	cyclin-dependent kinase 1
Nkx6-1	NM_144955	0.239	NK6 homeobox 1
Fam83d	NM_027975	0.247	family with sequence similarity 83, member D

Supplementary Table V. Gene expression patterns in the extracellular matrix category after rotator cuff tear (a, week 1 after tear; b, week 4 after tear)

a)

Gene symbol	Genbank accession	Fold change	Gene name
Upregulated			
Il1rl1	NM_010743	64.484	interleukin 1 receptor-like 1
Fbn2	NM_010181	63.172	fibrillin 2
Slpi	NM_011414	47.775	secretory leukocyte peptidase inhibitor
Lgals3	NM_001145953	35.562	lectin, galactose binding, soluble 3
Cthrc1	NM_026778	34.829	collagen triple helix repeat containing 1
Runx1	NM_001111023	32.822	runt related transcription factor 1
Hpse	NM_152803	26.165	heparanase
Adamts17	NM_001033877	22.069	a disintegrin-like and metallopeptidase (reprolysin type) with thrombospondin type 1 motif, 17
Mmp3	NM_010809	20.014	matrix metallopeptidase 3
Ccbe1	NM_178793	19.573	collagen and calcium binding EGF domains 1
Downregulated			
Apoh	NM_013475	0.092	apolipoprotein H
Adamts8	NM_013906	0.096	a disintegrin-like and metallopeptidase (reprolysin type) with thrombospondin type 1 motif, 8
Casp14	NM_009809	0.099	caspase 14
Egflam	NM_001289496	0.099	EGF-like, fibronectin type III and laminin G domains
Emid1	NM_080595	0.103	EMI domain containing 1
Timp4	NM_080639	0.111	tissue inhibitor of metalloproteinase 4
Myoc	NM_010865	0.111	myocilin
Wnt4	NM_009523	0.114	wingless-type MMTV integration site family, member 4
Chad	NM_007689	0.169	chondroadherin
Nepn	NM_025684	0.227	nephrocan

b)

Gene symbol	Genbank accession	Fold change	Gene name
Upregulated			
Mmp3	NM_010809	11.256	matrix metallopeptidase 3
Col6a5	NM_001167923	8.074	collagen, type VI, alpha 5
Crtac1	NM_145123	7.089	cartilage acidic protein 1
Runx1	NM_001111023	5.983	runt related transcription factor 1
Wnt3	NM_009521	5.407	wingless-type MMTV integration site family, member 3
Cilp	NM_173385	5.013	cartilage intermediate layer protein, nucleotide pyrophosphohydrolase
Col10a1	NM_009925	4.936	collagen, type X, alpha 1
Dsg1a	NM_010079	4.384	desmoglein 1 alpha
Lgals3	NM_001145953	4.043	lectin, galactose binding, soluble 3
Adamts16	NM_172053	3.816	a disintegrin-like and metallopeptidase (reprolysin type) with thrombospondin type 1 motif, 16
Downregulated			
Odam	NM_027128	0.077	odontogenic, ameloblast asssociated
Apoh	NM_013475	0.13	apolipoprotein H
Ltbp1	AK053964	0.136	latent transforming growth factor beta binding protein 1
Zg16	NM_026918	0.208	zymogen granule protein 16
Col6a4	NM_026763	0.3	collagen, type VI, alpha 4
Ntn1	NM_008744	0.313	netrin 1
Tnfrsf11b	NM_008764	0.35	tumor necrosis factor receptor superfamily, member 11b (osteoprotegerin)
Kazald1	NM_178929	0.387	Kazal-type serine peptidase inhibitor domain 1
Wnt4	NM_009523	0.39	wingless-type MMTV integration site family, member 4
Emid1	NM_080595	0.393	EMI domain containing 1

Supplementary Table VI. Gene expression patterns in the cell differentiation category after rotator cuff tear (a, week 1 after tear; b, week 4 after tear)

a)

Gene symbol	Genbank accession	Fold change	Gene name
Upregulated			
Cd300lf	NM_001169153	128.186	CD300 antigen like family member F
Krt8	AK166854	104.209	keratin 8
Rspo2	NM_172815	81.152	R-spondin 2 homolog (*Xenopus laevis*)
Clec4e	NM_019948	75.118	C-type lectin domain family 4, member e
Gpnmb	NM_053110	64.899	glycoprotein (transmembrane) nmb
Ccl3	NM_011337	60.502	chemokine (C-C motif) ligand 3
Trem2	NM_001272078	59.669	triggering receptor expressed on myeloid cells 2
Ccl12	NM_011331	59.221	chemokine (C-C motif) ligand 12
Clec4d	NM_010819	54.75	C-type lectin domain family 4, member d
Sox11	NM_009234	50.852	SRY (sex determining region Y)-box 11
Downregulated			
Foxb1	NM_022378	0.01	forkhead box B1
Pitx1	NM_011097	0.025	paired-like homeodomain transcription factor 1
Trpm1	NM_001039104	0.046	transient receptor potential cation channel, subfamily M, member 1
Gata4	NM_008092	0.05	GATA binding protein 4
Tbata	NM_023064	0.051	thymus, brain and testes associated
Fgf6	NM_010204	0.055	fibroblast growth factor 6
Nhlh2	NM_178777	0.056	nescient helix loop helix 2
Isl1	NM_021459	0.06	ISL1 transcription factor, LIM/homeodomain
Arhgef7	AK139156	0.062	Rho guanine nucleotide exchange factor (GEF7)
Brinp2	NM_207583	0.064	bone morphogenic protein/retinoic acid inducible neural-specific 2

b)

Gene symbol	Genbank accession	Fold change	Gene name
Upregulated			
Krt8	AK166854	16.55	keratin 8
Epha1	NM_023580	15.534	Eph receptor A1
Nr5a2	NM_030676	15.529	nuclear receptor subfamily 5, group A, member 2
Prss42	NM_153099	15.131	protease, serine 42
Krt17	NM_010663	14.697	keratin 17
Nrg1	NM_178591	13.811	neuregulin 1
Slc4a10	NM_001242380	13.099	solute carrier family 4, sodium bicarbonate cotransporter-like, member 10
Atp8b1	NM_001001488	12.571	ATPase, class I, type 8B, member 1
Nrcam	NM_176930	12.289	neuronal cell adhesion molecule
Myog	NM_031189	11.945	myogenin
Downregulated			
Rab25	NM_016899	0.022	RAB25, member RAS oncogene family
Nppa	NM_008725	0.085	natriuretic peptide type A
Pitx1	NM_011097	0.087	paired-like homeodomain transcription factor 1
Dct	NM_010024	0.108	dopachrome tautomerase
5830411N06Rik	NM_175533	0.114	RIKEN cDNA 5830411N06 gene
Ahi1	NM_026203	0.118	Abelson helper integration site 1
Csf2	NM_009969	0.122	colony stimulating factor 2 (granulocyte-macrophage)
Tlk2	NM_001294331	0.132	tousled-like kinase 2 (*Arabidopsis*)
Sprr2b	NM_011469	0.133	small proline-rich protein 2B
Plk4	NM_011495	0.146	polo-like kinase 4
